# Pathophysiology of REM sleep behaviour disorder

**DOI:** 10.1093/braincomms/fcag388

**Published:** 2026-10-15

**Authors:** Nita E Alpin, Kristijan D Jovanoski, Pietro-Luca Ratti, David A Menassa, Laura Parkkinen

**Affiliations:** Nuffield Department of Clinical Neurosciences, University of Oxford, Oxford OX3 9DU, United Kingdom; Nuffield Department of Clinical Neurosciences, University of Oxford, Oxford OX3 9DU, United Kingdom; Nuffield Department of Clinical Neurosciences, University of Oxford, Oxford OX3 9DU, United Kingdom; Department of Clinical Neurophysiology, Oxford University Hospitals NHS Foundation Trust, Oxford OX3 9DU, United Kingdom; Nuffield Department of Clinical Neurosciences, University of Oxford, Oxford OX3 9DU, United Kingdom; Department of Women’s & Children’s Health, Karolinska Institutet, Solna SE-171 77, Sweden; Nuffield Department of Clinical Neurosciences, University of Oxford, Oxford OX3 9DU, United Kingdom

**Keywords:** REM sleep behaviour disorder, synucleinopathy, neuropathology, neurodegenerative disease

## Abstract

REM sleep behaviour disorder (RBD) is a parasomnia characterized by loss of muscle atonia and dream enactment behaviour during REM sleep. RBD is recognized as a significant prodromal marker of α-synucleinopathies, but characterizing its unique neuropathological features is challenging due to reliance on individual case reports. Additionally, RBD is not a terminal illness, and data generalisability is limited by the high co-occurrence of α-synucleinopathies. In this review, we discuss the mechanisms responsible for altered REM sleep regulation in RBD, incorporating evidence from human post-mortem histological analyses, structural and functional neuroimaging data on patients with RBD, and pharmacological studies. We explore the risks, biomarkers, and mechanisms of phenoconversion from RBD to α-synucleinopathies, drawing on data from large longitudinal patient cohorts, and we consider disease remission. Lastly, we propose several promising directions for future studies.

## Graphical Abstract

**Graphical Abstract:**
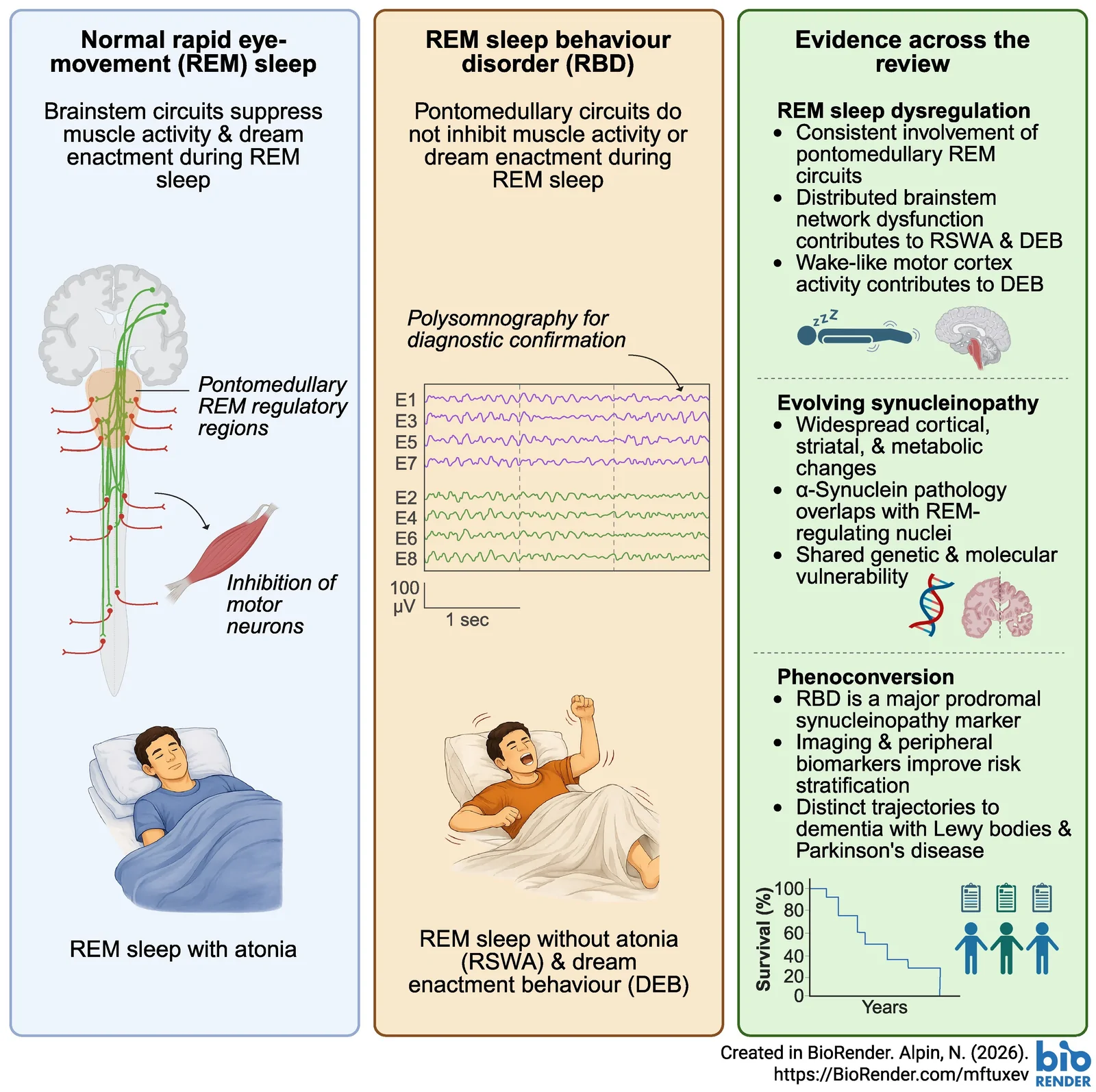

## Teaser

Alpin *et al*. review evidence for the pathophysiology of rapid eye movement (REM) sleep behaviour disorder, concluding that distributed brainstem network dysfunction underlies REM sleep dysregulation, while broader cortical, striatal, and metabolic changes largely reflect evolving neurodegeneration and progression to Parkinson’s disease and dementia with Lewy bodies.

## Introduction

α-Synucleinopathies are a clinically and pathologically heterogenous group of neurodegenerative disorders characterized by the presence of fibrillar aggregates of α-synuclein in nerve fibres and glial cells, and of Lewy bodies (LBs) in neuronal somata.^1,2^ These disorders include Parkinson’s disease (PD), Parkinson’s disease dementia (PDD), dementia with Lewy bodies (DLB), and multiple system atrophy (MSA). α-Synucleinopathies generally differ in their underlying cellular pathology, affected brain regions, and subsequent clinical presentation,^3,4^ which feature various combinations of symptoms such as motor impairment, autonomic dysfunction, cognitive impairment, and sleep disturbances.

Classical symptoms of α-synucleinopathies may emerge only two decades after the onset of protein aggregation in neurons and glia and neurodegeneration,^5–7^ with clinically significant sleep disturbances, such as REM sleep behaviour disorder (RBD, Box 1 and Table 1), often occurring earlier in the disease course.^28^ RBD manifests as REM sleep without atonia (RSWA) and dream enactment behaviour (DEB) during REM sleep,^8^ and is a major prodromal marker of an α-synucleinopathy. However, whether RBD reflects a primary driver of synucleinopathy progression, or an early or parallel manifestation of selective network vulnerability, remains unsolved. According to Braak’s staging system of α-synuclein pathology,^29^ LBs first emerge in medullary and olfactory nuclei (Stage 1) before appearing in more rostral areas such as the pontine tegmentum (Stage 2), midbrain (Stage 3), amygdala and limbic structures (Stage 4), and neocortical areas (Stages 5 and 6). Individuals with isolated RBD may therefore exhibit an early clinical manifestation of a neurodegenerative process. Moreover, post-mortem histopathological analyses on isolated RBD show CNS α-synuclein deposits consistent with synucleinopathies^10–12^ and seed amplification assays (SAAs) can detect phosphorylated α-synuclein in RBD biosamples such as CSF and skin biopsies.^30–32^

Box 1 REM sleep behaviour disorder

RBD is a parasomnia characterized by the loss of skeletal muscle atonia during REM sleep (REM sleep without atonia, RSWA), with prominent motor activity concurrent with dreaming.^8^ While the classification of RBD as an isolated syndrome is debated due to its strong association with neurodegenerative diseases, RBD is termed *isolated* (formerly ‘idiopathic’) if it occurs in absence of any overt neurological disorder, and *secondary* if it is associated with one. 66% of individuals with RBD begin showing symptoms related to α-synucleinopathies within 7.5 years from initial symptom onset.^9^ In the few isolated RBD cases that underwent autopsy, histopathological evaluation showed neuronal α-synuclein accumulation in the form of LBs and Lewy neurites representative of Braak LB stages 3 to 5.^9–12^ Imaging studies have also shown impaired dopaminergic signalling in individuals with RBD despite an absence of motor symptoms, suggesting that RBD may serve as a prodromal marker of α-synucleinopathies.^13–15^

Clinically, an RBD diagnosis requires polysomnographic (PSG) confirmation of both RSWA and sleep-related behaviours that are injurious, potentially injurious, or disruptive (i.e. DEB). These behaviours can be observed by either the patient or their bed partner or documented during PSG monitoring^16^ (Table 1). Behaviours that are characteristic of RBD must manifest during the REM sleep stage, distinguishing RBD from non-REM (NREM) parasomnias such as sleepwalking and non-pathological dream enactment occurring upon arousals from REM or NREM sleep. Incorporating PSG monitoring in research studies is therefore fundamental, as PSG is essential for diagnosing RBD in an individual with abnormal sleep-related behaviours.^26,27^

While clinical cohorts show a male predominance in RBD,^17,18^ well-designed population studies do not indicate a sex difference.^19^ Male patients may seek medical assistance and receive sleep clinic referrals more rapidly, likely due to more violent or injurious dream enactment.^20,21^ This bias is also present in the neuropathology literature, in which female cases are markedly under represented. In the absence of brainstem lesions, RBD co-occurs most often with α-synucleinopathies or narcolepsy, with prevalences ranging from 25% to 58% in PD, 70% to 80% in DLB, 90% to 100% in MSA,^8,22–24^ and 7% to 63% in narcolepsy.^25^

**Table 1:** International classification of sleep disorders (ICSD-3-TR) diagnostic criteria for RBD

Clinical feature	Measurement	Manifestation
REM sleep without atonia (RSWA)	Polysomnography (PSG)	Persistent or intermittent elevation of muscle tone in the EMG channel
Dream enactment behaviour (DEB)	Video monitoring during PSG	Injurious, potentially injurious, or disruptive behaviours
	History from bedpartner/family	Reports of recurrent injurious, potentially injurious, or disruptive behaviours and/or of vocalisations in keeping with the enactment of a dream

Population-based studies estimate the prevalence of RBD to be at ∼1% in adults over 50,^19^ contrasting sharply with its high predictive value for neurodegenerative disease. Approximately 60% of individuals with RBD go on to develop PD,^33^ and up to 90% will eventually progress to some form of α-synucleinopathy.^34^ The association between RBD and neurodegenerative diseases is therefore well established.^35–38^ However, RBD is by no means 100% predictive of a developing α-synucleinopathy. RBD onset can occur prior to^34,39–42^ or following a diagnosis of PD or another α-synucleinopathy.^43,44^ RBD has also been described as a comorbidity in autoimmune diseases such as narcolepsy,^25^ limbic encephalitis,^45^ and multiple sclerosis (MS),^46,47^ as well as in patients with brainstem lesions^48^ and in association with antidepressant use.^49–51^ This comorbid occurrence with other diseases and conditions may help to elucidate the associated mechanisms and neuropathology of RBD in synucleinopathies.

Recent advances in the synucleinopathy-field have introduced the body-first versus brain-first hypothesis, which proposes that the initial site of α-synuclein pathology in synucleinopathies may determine the prodromal features and clinical progression of disease.^52–54^ In body-first cases, pathology is hypothesized to ascend from the peripheral autonomic nervous system through the brainstem, whereas the brain-first subtype may present with initial pathology in the olfactory areas and amygdalae. RBD as a prodromal marker is increasingly recognized as a hallmark of the body-first subtype, with phosphorylated α-synuclein found in colonic biopsies.^55,56^ Therefore, REM-regulating brainstem networks may represent selectively vulnerable entry points for body-first α-synuclein pathology in at least a subset of individuals with RBD. When synuclein pathology initiates in the rostral brain areas and descends to brainstem later, RBD may be preceded with cognitive and psychiatric symptoms and would not necessarily present prodromally. This variability in the relative timepoint of brainstem involvement may help explain the heterogeneity in symptom onset and disease progression observed in RBD cohorts.

Despite the strong epidemiological link between RBD and α-synucleinopathies, several critical gaps remain in our understanding of its pathophysiology. Existing literature is fragmented and often derives mechanistic insights from small post-mortem series, lesion studies, neuroimaging, and pharmacological observations that are frequently interpreted in isolation. A major challenge in defining mechanisms directly responsible for RBD is that isolated RBD cohorts frequently already exhibit changes associated with an evolving synucleinopathy. Previous reviews of RBD have therefore largely focused on its clinical utility as a prodromal marker^22,57,58^ or on modality-specific findings^59,60^ to derive mechanistic insight. Such a focus may overlook mechanisms that are specific to REM sleep dysregulation or relevant to the clinical heterogeneity of RBD itself.

Here, we organize the literature to distinguish mechanisms potentially related to REM dysregulation from abnormalities that may reflect broader phenoconversion trajectories and evolving neurodegeneration. Within this framework, alterations in pontomedullary REM-regulatory circuits may contribute more directly to RSWA and DEB, whereas widespread cortical, striatal, metabolic, and connectivity abnormalities may reflect broader phenoconversion trajectories, disease subtype, compensatory responses, and parallel neurodegenerative processes. Rather than proposing a single explanatory model, we seek to clarify areas of convergence and uncertainty across neuropathology, neuroimaging, pharmacology, genetics, and biomarker research. With a mechanistic focus on REM sleep regulation, we also seek to reconcile why many identified abnormalities in RBD overlap with established α-synucleinopathies.

Specifically, we explore how dysfunction within REM sleep-regulating brainstem networks in RBD relates to selective neuronal vulnerability and phenoconversion to α-synucleinopathies. To address mechanisms of selective vulnerability, we first discuss the human systems responsible for normal REM sleep control, the known neuropathological correlates of RBD, and their relationship to other neurological and neurodegenerative diseases. We then focus on phenoconversion, summarizing the risks, biomarkers, and proposed mechanisms of differential progression to PD and DLB. Throughout the review, we discuss factors that may modulate disease expression and progression, as well as methodological limitations and conceptual questions that remain unresolved. Lastly, we propose future directions that may help guide research on the pathophysiology of RBD and its phenoconversion to neurodegenerative disease.

## Search strategy

This narrative review was informed by a structured PubMed literature search for studies published in English before 26 May 2026. Studies were prioritized according to relevance to human polysomnography (PSG)-confirmed RBD, with emphasis on longitudinal multicentre cohorts, prospective studies, and investigations with neuropathological or biomarker confirmation where available. Review articles were used to contextualize findings and identify primary sources. Where conflicting findings existed, greater weight was given to longitudinal multicentre cohorts, PSG-confirmed RBD, pathology-supported diagnoses, and studies with replication across modalities. Case reports and small post-mortem series were interpreted primarily for hypothesis-generating insights. Search terms included combinations of ‘*REM sleep behaviour disorder*’ or ‘*RBD*’ or ‘*REM sleep*’ with ‘*neuropathology*’, ‘*neuroimaging*’, ‘*MRI*’, ‘*fMRI*’, ‘*EEG*’, ‘*diffusion imaging*’, ‘*FDG-PET*’, ‘*DAT-SPECT*’, ‘*treatment*’, ‘*levodopa*’, ‘*melatonin*’, ‘*cholinesterase inhibitors*’, ‘*rivastigmine*’, ‘*donepezil*’, ‘*benzodiazepine*’, ‘*clonazepam*’, ‘*GWAS*’, ‘*seed amplification assay*’ or ‘*SAA*’ or ‘*RT-QuIC*’, ‘*biomarker*’, ‘*conversion*’, ‘*phenoconversion*’, ‘*progression*’, ‘*remission*’, ‘*alpha-synuclein*’ or ‘*a-synuclein*’, ‘*synucleinopathy*’ or ‘*a-synucleinopathy*’, ‘*Parkinson’s disease*’ or ‘*PD*’, ‘*Parkinson’s disease dementia*’ or ‘*PDD*’, ‘*dementia with Lewy bodies*’ or ‘*DLB*’, ‘*Lewy body disease*’ or ‘*LBD*’, and ‘*Multiple system atrophy*’ or ‘*MSA*’.

## REM sleep control and RBD

First, we need to understand the sleep control points whose failure may be associated with RBD. Sleep is broadly divided into two phases: non-REM (NREM) sleep, which consists of three progressively deeper stages (N1, N2, and N3), and REM sleep (Fig. 1A). Normal REM sleep is characterized by unique physiological and behavioural features, including waking-state-like low amplitude and desynchronized scalp EEG activity, skeletal muscle paralysis, and the activation of autonomic and respiratory systems.^61^ The core neural circuitry governing REM sleep is in the dorsal brainstem and is critical for the generation of both muscle atonia and the cortical features of REM sleep.^62^ Given that RBD is the focus of this review and RSWA is a core component of the disorder, we will specifically focus on the mechanisms and neuronal pathways involved in generating skeletal muscle paralysis and inhibiting DEB.

**Figure 1:**
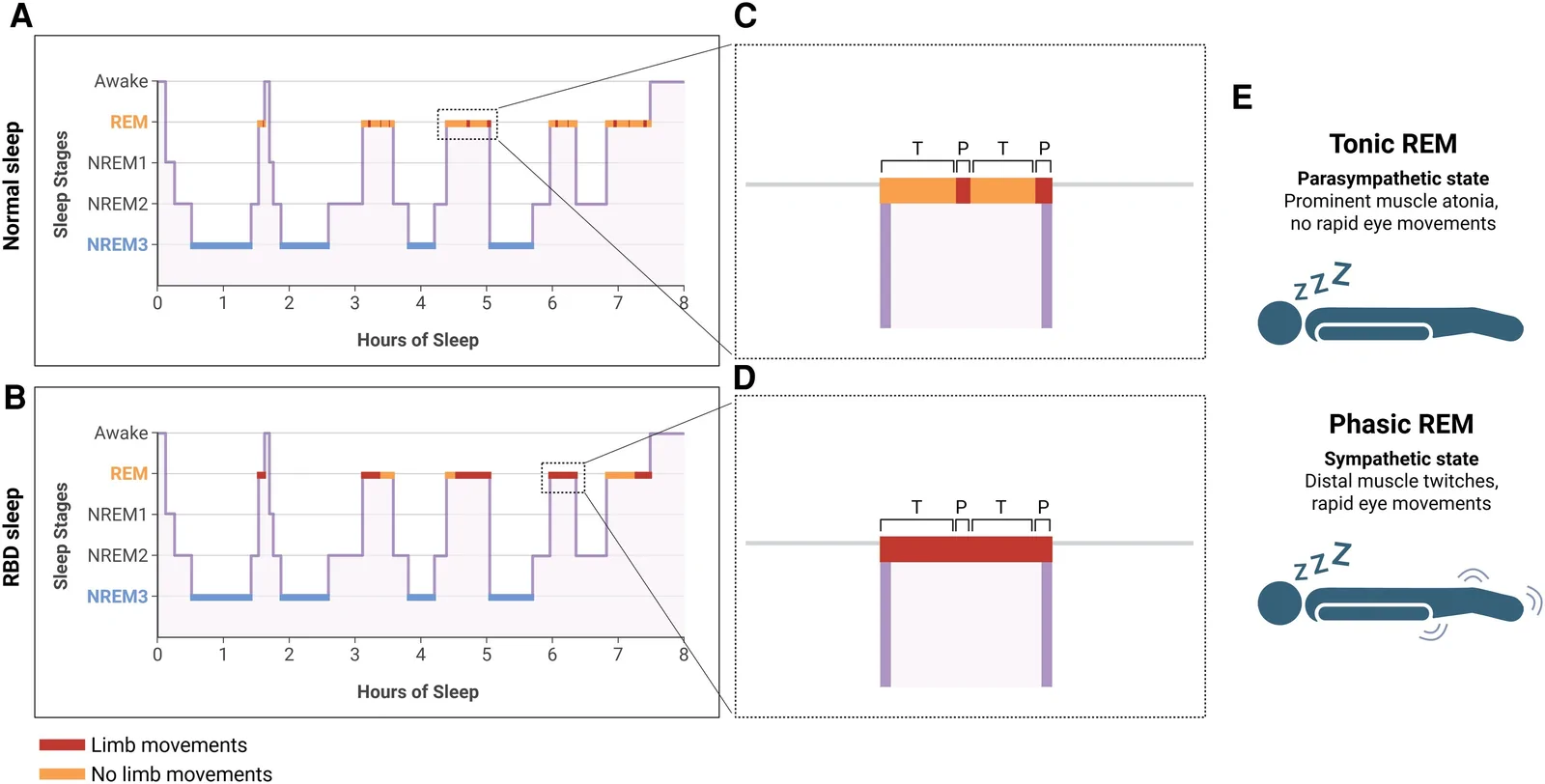
**Comparison of sleep architecture and REM microstates in normal sleep and RBD.** Illustration of the progression of sleep stages across an 8-hour period for a typical night of normal sleep (**A**) and RBD sleep (**B**). REM periods are highlighted, with superimposed bars indicating the presence of limb movements. Magnified views of a representative REM episode from normal sleep (**C**) and RBD sleep (**D**), showing the alternation between tonic (T) and phasic (P) R**E**M microstates. In normal sleep (**C**), limb movements are infrequent and primarily coincide with phasic REM. In RBD (**D**), muscle activity is more frequent and can occur during both tonic and phasic REM. (**E**) Illustration highlighting sustained muscle atonia during tonic REM and distal muscle twitches during phasic REM. Created in BioRender. Alpin, N. (2026) https://BioRender.com/ueg6xgi.

### Neurophysiology of REM sleep

The structure of REM sleep and its breakdown is thought to be important for the emergence of RSWA and DEB in RBD, although the current evidence base remains relatively limited. While REM sleep is often described as a homogenous state, it consists of alternating tonic and phasic microstates.^63^ Tonic REM is characterized by parasympathetic predominance, marked muscle atonia, and the absence of eye movements, and constitutes ∼80–90% of REM sleep time.^64^ In contrast, phasic REM is dominated by sympathetic activation and bursts of rapid eye movements and distal muscle twitches that last from a few seconds to a few minutes at a time^64^ (Fig. 1A and B).

Since DEB can be exhibited during both microstates, some studies suggest that the distinction between tonic and phasic REM sleep may be reduced in RBD^65–68^ (Fig. 1D and E). High density scalp EEG recordings have demonstrated reduced beta activity during phasic REM sleep in RBD.^69^ Since beta activity is generally associated with cortical arousal and wake-like vigilance, reduced beta power during phasic REM could reflect altered arousal regulation or impaired gating of dream content. However, the functional significance of these findings remains uncertain, and current evidence is insufficient to determine whether disruptions in REM microstate regulation are central mechanisms of RBD. We also do not know whether the extent of microstructure alterations translates to the extent of general REM sleep dysregulation.

Altered REM microstructure may provide one framework for understanding the pathophysiology of RBD. However, while RSWA is a key clinical feature of RBD, pharmacological effects, intense dream content, and transient mixed-sleep states can also evoke DEB.^70^ Since DEB is also observed in conditions with sleep-state dissociation, DEB may not be exclusive to phasic REM or even REM sleep in general.^70^

Individuals with PD and comorbid RBD often display relatively normal motor function during DEB,^71^ which contrasts sharply with the bradykinesia they experience while awake. This observation has led to the hypothesis that motor cortex activity during REM sleep may partially bypass the pathological cortico-basal ganglia circuitry and engage alternative central motor pathways.^72–75^ EEG studies have identified abnormal motor cortex activity in RBD during REM sleep that resembles the EEG patterns of voluntary movements during wakefulness.^75^ Whether these electrophysiological changes represent a primary disturbance of REM-state regulation or simply reflect downstream consequences of impaired brainstem inhibitory control as the principal mechanism underlying DEB remains unclear.^66^

### Brainstem structures involved in REM sleep control in RBD

Altered REM microstate alternation has been proposed as one possible contributor to RSWA and may reflect the early dysfunction of pontomedullary REM-regulating networks that are selectively vulnerable in α-synucleinopathies. However, complex DEB is more likely to arise from multilevel engagement of circuits across the CNS.^76^

Our understanding of brainstem structures implicated in dysregulated REM sleep control (Fig. 2) in RBD has evolved over time. When Boeve *et al.*^77^ proposed an anatomical framework for RBD in 2007, many brainstem structures involved in normal human REM sleep control had not yet been fully identified and were assumed comparable to those described in animal studies. Early studies in the cat suggested that REM sleep is controlled by two motor systems, one responsible for the inhibition of lower motor neurons and the other for inhibiting central locomotor generators.^78–82^

**Figure 2:**
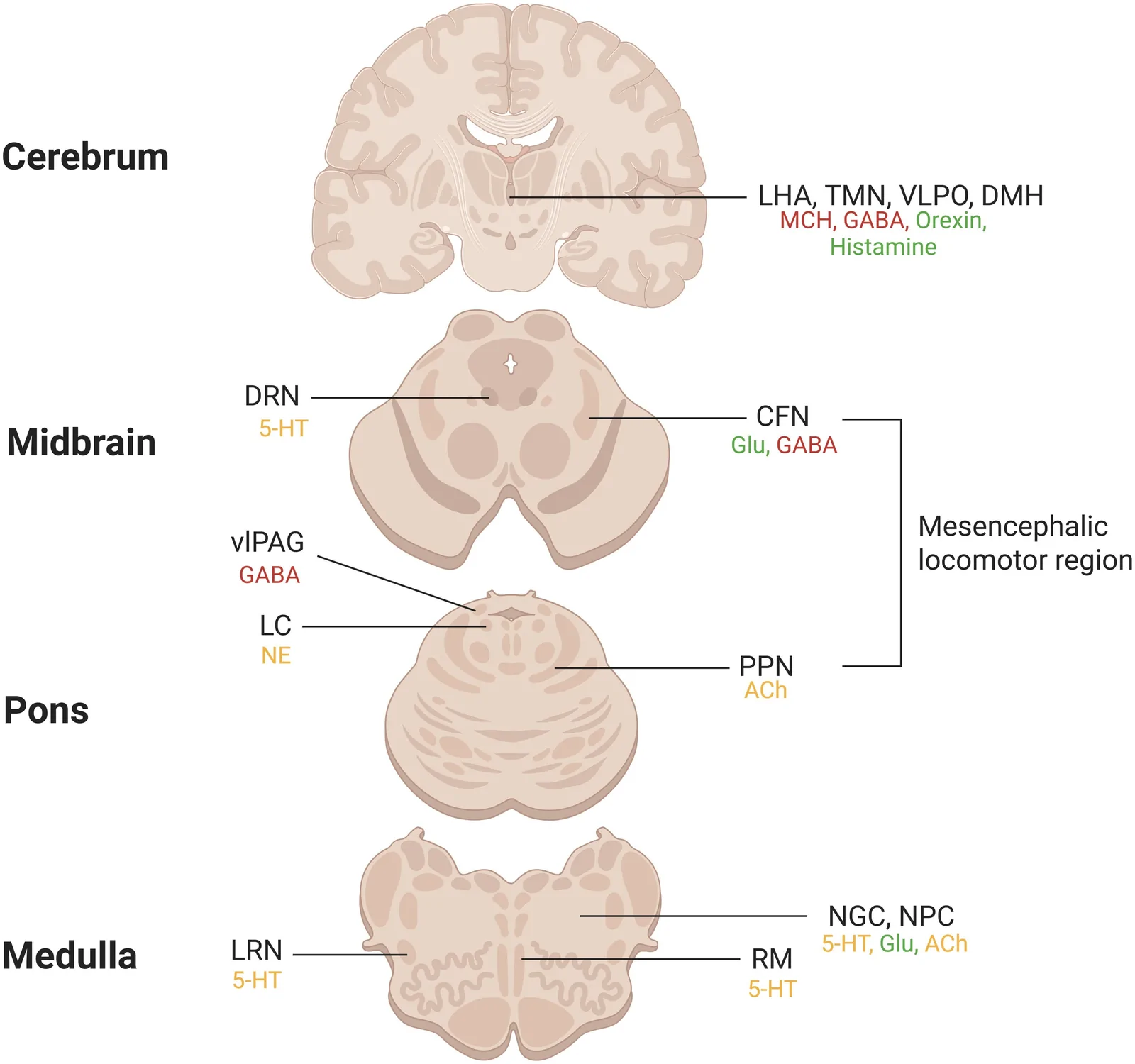
**Nuclei and chemical messengers associated with REM sleep control.** Anatomical abbreviations: CFN, cuneiform nucleus; DMH, dorsomedial hypothalamus; DRN, dorsal raphe nucleus; LC, locus coeruleus; LHA, lateral hypothalamic area; LRN, lateral raphe nucleus; NGC, nucleus gigantocellularis; NPC, nucleus parvocellularis; PPN, pedunculopontine nucleus; RM, raphe magnus; TMN, tuberomammillary nucleus; vlPAG, ventrolateral periaqueductal grey; VLPO, ventrolateral preoptic area. Chemical messenger abbreviations: 5-HT, 5-hydroxytryptamine (serotonin); ACh, acetylcholine; Glu, glutamate; MCH, melanin-concentrating hormone; NE, norepinephrine. Chemical messengers can have inhibitory (MCH, GABA), excitatory (Orexin, Histamine, Glu), or mixed (5-HT, NE, ACh) effects on target nuclei. Created in BioRender. Alpin, N. (2026) https://BioRender.com/nk9krlz.

Combining these findings with more recent studies in the rat, Boeve *et al.*^77^ postulated that the magnocellular reticular formation (MCRF), spanning the pons and medulla and modulated by pontine nuclei such as the norepinephrinergic locus coeruleus (LC)-subcoeruleus (SubC) complex, cholinergic pedunculopontine nucleus (PPN), and periaqueductal grey (PAG), would directly activate spinal interneurons, resulting in muscle atonia. Given that some individuals demonstrate PSG evidence of RSWA but have never exhibited DEB, the authors hypothesized that muscle atonia and DEB would be controlled by distinct networks. This distinction remains influential, although accumulating evidence suggests that RSWA and DEB may partially dissociate and reflect overlapping rather than entirely discrete network dysfunctions. Of note, some individuals demonstrate substantial RSWA with minimal or absent DEB,^83,84^ and both RSWA and DEB frequency can fluctuate night-to-night.^85^

Recent studies suggest that the systems controlling REM sleep (Fig. 2) may span more rostrally and include the glutamatergic and GABAergic cuneiform nucleus (CFN), which works in concert with the PPN to initiate and modulate spinal neural circuitry.^86,87^ Together, the CFN and PPN form the mesencephalic locomotor region (MLR). Extensive experiments in decerebrate cats show that the activation of PPN neurons suppresses skeletal muscle tone via cholinergic projections to the reticular formation, while the activation of CFN elicits locomotion.^88–92^ PPN neurons have been shown to not only control the level of muscle tone but also modulate the locomotor pattern at the pontine level,^89,90^ suggesting that the nucleus has a key dual role in regulating both functions.

Although the PPN is actively involved in promoting REM sleep via the thalamocortical network, its ascending and descending projections are inconsistent. Moreover, the PPN has numerous auxiliary loops within corticobasal circuitry, indicating that it is not a major output structure.^93^ For example, high-frequency PPN deep brain stimulation (DBS) during wakefulness has been shown to increase the feeling of sleepiness, induce non-REM sleep,^94^ and improve both the time and quality of REM sleep in PD patients.^95^ However, PPN-DBS does not result in appreciable differences in motor activity during REM sleep in patients with comorbid RBD,^95^ suggesting that the PPN alone may not be the primary controller of muscle atonia during REM sleep. Since the PPN is a heterogeneous nucleus, the specific cell populations targeted by DBS remain unidentified. While stimulating specific PPN subpopulations may still induce muscle atonia, it is possible that these subpopulations may already be too extensively compromised in individuals with RBD to produce an effect upon stimulation. More broadly, REM motor control is likely mediated through distributed circuits, making it difficult to attribute RBD pathophysiology to a single neurotransmitter system or anatomical structure.

Taken together, the more recent studies challenge the earlier hypothesis that RSWA and DEB result from completely independent pathological processes. Nevertheless, RSWA appears to be more related to pontomedullary inhibitory pathways,^96,97^ whereas complex DEB likely requires additional disinhibition of locomotor and cortico-limbic networks.^98^ In an integrated view, selective disruption of the MLR could generate either RSWA, DEB, or both, depending on whether inhibitory relays, locomotor drives, or their gating connections are affected.

Other structures involved in REM sleep control (Fig. 2) include the dorsal raphe nucleus (DRN) in the midbrain, the ventrolateral PAG (vlPAG) and LC in the pons, and the lateral raphe nucleus (LRN), raphe magnus (RM), nucleus gigantocellularis (NGC), and nucleus parvocellularis (NPC) in the medulla. The subcoeruleus (SubC) has also been suggested to play a role in the circuitry (Box 2). Current evidence supports a hierarchically organized network of reciprocal connections in which pontine areas recruit medullary inhibition of atonia, while mesencephalic locomotor areas and monoaminergic nuclei shape the expression and timing of REM sleep.^62^ However, the relative contribution of individual nuclei to RSWA and DEB likely varies across clinical phenotype and disease stage.

Box 2 The subcoeruleus (SubC) region

Of the brainstem structures implicated in REM sleep regulation, the SubC region is thought to play a key role, particularly in generating motor atonia via inhibitory GABAergic and glycinergic neurons in the ventromedial medulla and spinal cord.^62^ However, in the human brain, its precise anatomical location and relevance remain unresolved. While animal studies often equate the SubC with structures such as the sublaterodorsal nucleus (SLD) in rodents and peri-LCα in cats,^96^ human neuroanatomy literature lacks consensus on the existence or clear delineation of the SubC. Brain atlases further complicate this picture, as the Allen Human Brain Atlas recognizes the SubC as a distinct nucleus slightly dorsocaudal to the LC with its own molecular signatures [e.g. higher vasoactive intestinal peptide (VIP) expression],^99^ while other studies describe the SubC as an ill-defined area either fully or partially adjacent to^77,100^ or caudal to^101^ the LC, extending well into the caudal pons.

Discrepancies in SubC location across studies are likely to reflect differences in brainstem orientation and section plane descriptions rather than true anatomical disagreement. Consequently, the α-synucleinopathy-related neuropathological and imaging studies discussed in the following sections often assess α-synuclein deposits and neuromelanin reactivity across the LC-SubC complex without (explicitly) distinguishing its finer subdivisions.^11,12,102,103^ Increased availability of the whole pons in brain banks and refinement of neuromelanin-sensitive MRI techniques may help to distinguish the LC and the SubC and elucidate their involvement in RSWA and DEB.

The network is modulated by hypothalamic innervations, which supply the circuit with circadian rhythmicity. Under normal REM sleep, hypothalamic innervations to the LC inhibit its function and it falls silent, thereby allowing the GABAergic ventromedial medulla and spinal interneurons to hyperpolarize motor neurons, facilitating muscle atonia.^96^ While reduced LC activity is necessary for sleep initiation, it is primarily involved in wakefulness regulation, with its intermittent activity during sleep playing a modulatory role.^104^

## The neuropathology of RBD

Despite our understanding of brainstem structures involved in REM sleep control, we do not yet understand how pathology in the brainstem specifically leads to RBD. Since RBD is strongly associated with neurological and neurodegenerative diseases, identifying a neuropathological signature specific to RBD is challenging and relies heavily on rare case reports and small patient cohorts which are often already undergoing α-synucleinopathy-related changes. Moreover, even sufficiently large post-mortem cohorts are heterogeneous in the temporal sequence of diagnoses (i.e. whether RBD precedes, coincides with or follows the onset of other conditions). Despite these challenges, post-mortem, imaging, pharmacological, and genetic studies have provided valuable clues about RBD-specific pathophysiology.

### Post-mortem studies

#### Isolated RBD cases

Evidence regarding the neuropathological correlates of isolated RBD largely originates from post-mortem studies of patients with isolated RBD, i.e. without a concurrent neurological disorder (Box 1). However, these studies are extremely rare, and generally lack control groups of healthy patients without RBD. To date, only five autopsies have been performed on patients with putative isolated RBD, all of whom were male. All patients were diagnosed with an α-synucleinopathy upon post-mortem evaluation, although the degree of pathology and affected nuclei varied amongst cases.

##### Case 1

In 1995, Uchiyama *et al*.^10^ examined an 86-year-old patient with a 20-year history of violent DEB, but with no clinical signs of other neurological diseases up to his time of death from pneumonia. They observed severe degeneration of the substantia nigra (SN) and LC, with occasional LBs in the surviving neurons. LBs were also found in the DRN, central raphe nucleus (CRN, also known as the superior central nucleus), PPN, NGC, and ventral reticular nucleus, but these areas did not show degenerative changes. The development of α-synuclein antibodies in the late 1990s, followed by the publication of the Braak LB^29^ and McKeith^105^ staging systems in 2003 and 2005, respectively, have since enabled the characterization of this patient as having either Braak LB stage 3 PD or McKeith brainstem-predominant Lewy body disease (LBD). Given the involvement of the major monoamine-synthesising nuclei, SN and LC, Uchiyama *et al*.^10^ postulated the loss of monoaminergic neurons as a potential cause of RBD symptomology.

##### Case 2

Twelve years later, Boeve *et al.*^11^ presented a case of a 72-year-old PSG-confirmed RBD patient who later died from cryptogenic organizing pneumonia. Despite no other neurological symptoms, histological evaluation at post-mortem revealed pathology consistent with Braak LB stage 3 or McKeith brainstem-predominant LBD. In this case, the SN, LC, CRN, and PPN showed only minimal degenerative changes, challenging the view that monoaminergic loss or alterations to the PPN are the primary cause of RBD.

##### Cases 3, 4 and 5

The largest neuropathological series of individuals with isolated RBD was published in 2024 by Mayà *et al.*^12^ and included three cases: a 60-year-old male (sudden death), a 93-year-old male (respiratory infection), and a 78-year-old male (lung cancer). None of the patients exhibited other neurological symptoms, yet post-mortem histological assessment showed pathology consistent with MSA in the first case and LBD in the second (Braak LB stage 5) and third (Braak LB stage 4) cases. Mild-to-moderate cell loss was identified in brainstem structures involved in REM sleep atonia, including the NGC, LC-SubC complex, laterodorsal tegmentum, and SN. The study also noted minimal cortical involvement and a low burden of concomitant pathologies such as β-amyloid and tau. However, the absence of control subjects or quantitative assessments limits the ability to pinpoint the exact regions responsible for RBD symptoms.

These post-mortem findings highlight a consistent association between α-synuclein pathology in brainstem regions involved in REM sleep regulation, supporting the role of RBD as an early feature of α-synucleinopathies. However, variability in the degree of degeneration, particularly in the LC and PPN, suggests that these regions may play a modulatory rather than causal role in RBD pathogenesis. This underscores the complexity of brainstem circuit dysfunction in RBD. Moreover, it is not possible to distinguish between changes directly related to RBD and those associated with α-synuclein pathology at Braak LB stages 3–5.

#### Secondary RBD cases in patients with PD, DLB and MSA

Further insights into the neuropathology of RBD have been provided by research on non-isolated RBD. In 2013, Iranzo *et al.*^106^ conducted neuropathological examinations on three patients who died of myocardial infarction, acute stroke, and pneumonia, respectively. All patients had a history of RBD, and two of them had converted to PD and one to DLB prior to death. The authors noted moderate-to-severe neuronal loss, gliosis, LBs, and Lewy neurites in the SubC, NGC and PPN in all three patients.

In 2014, Iranzo *et al.*^107^ also published a case report on a 69-year-old man with a 2-year history of DEB who eventually developed the common premotor signs of PD and DLB, namely hyposmia, constipation, depression, and mild cognitive impairment (MCI). Neuropathological evaluation showed Lewy pathology and neuronal loss in the olfactory bulb, medulla, pons, SN (with an estimated cell loss of 20% to 30%), nucleus basalis of Meynert (NBM), and amygdala.

In another case reported by Schenck *et al.*,^108,109^ a 72-year-old man initially diagnosed with isolated RBD later developed Alzheimer’s disease. Neuropathological examination revealed a marked loss of LC neurons but a higher number of cholinergic PPN neurons than age-matched control subjects. The authors suggested that RBD may result from the simultaneous increase in cholinergic neurons and their possible disinhibition due to LC cell loss. However, another investigation on four MSA patients with comorbid RBD described a contradictory systematic and severe depletion of both LC and PPN neurons.^110^

While these findings suggest that RBD is a prodromal manifestation of neurodegeneration rather than an isolated syndrome, the varying results and comorbidities complicate the identification of the exact structures involved in RBD pathogenesis. Together with recent experimental evidence, these findings suggest that the role of the PPN in RBD is likely to be modulatory rather than central.^111^ Additionally, recent resting-state functional MRI (fMRI) findings indicate that pathology in the LC may be partially specific to RBD; isolated RBD patients and PD patients with comorbid RBD, but not patients with isolated PD, exhibit disrupted LC functional connectivity.^112^ However, given that the LC is silent during REM sleep while cholinergic activity increases, LC dysfunction alone is unlikely to be directly responsible for RBD.^104,113^

Collectively, post-mortem studies on isolated and secondary RBD support a model of distributed network vulnerability rather than a single primary site framework. The neuropathological findings converge on the involvement of LC-SubC complex, NGC, and other ponto-medullary regions (Table 2 and Fig. 3). However, the degree of neuronal loss and α-synuclein burden within individual nuclei, particularly the LC and PPN, varies substantially across cases. This variability suggests that RSWA and DEB likely result from dysfunction across interconnected inhibitory, locomotor, and modulatory brainstem networks rather than isolated neuronal loss or α-synuclein accumulation in individual nuclei. Importantly, many of the affected nuclei participate in broader cognitive, autonomic, and motor systems and are also affected in α-synucleinopathies. The findings may thus help explain the heterogeneity of phenoconversion trajectories in RBD but they do not fully explain the primary mechanisms underlying RBD. Moreover, the inherently small sample sizes, frequent presence of comorbid neurodegenerative or systemic disease, marked underrepresentation of female cases, and the near-absence of age-matched and sex-matched control brains without RBD warrant caution in the interpretation of post-mortem findings. Given that population-based studies do not indicate a clear sex difference in RBD prevalence, the male predominance in neuropathological literature may limit the generalisability of current findings across sexes. Although the contribution of post-mortem studies to our understanding RBD pathophysiology is invaluable, case-based neuropathological data should be interpreted as a platform for hypothesis-generation.

**Figure 3:**
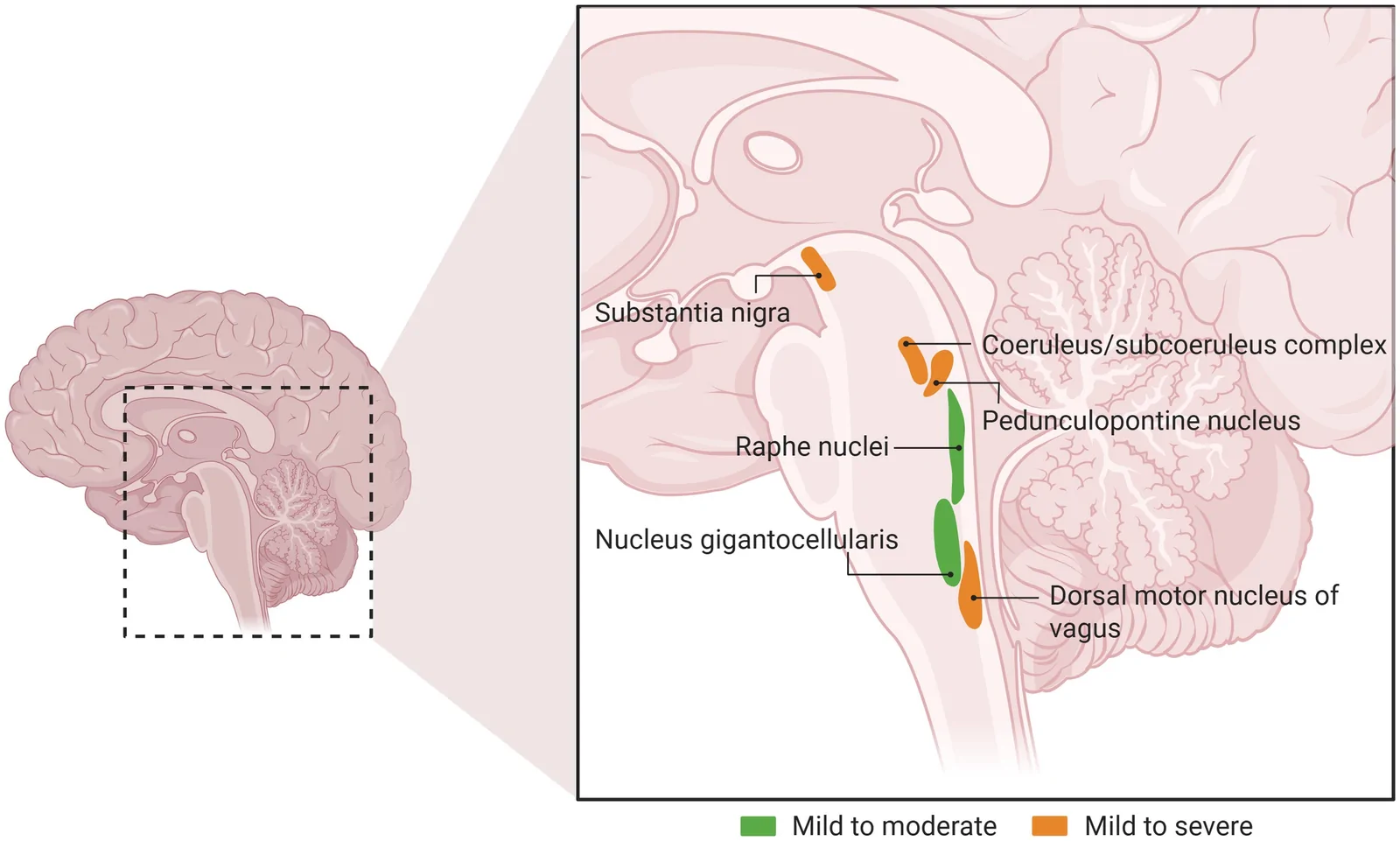
**Summary of the brainstem nuclei potentially involved in RBD symptomology based on post-mortem studies.** Brainstem diagram highlighting the key nuclei involved in RBD symptomology based on post-mortem analyses of both isolated RBD and secondary RBD. The severity of pathology per region varies from mild to moderate (raphe nuclei, nucleus gigantocellularis) and from mild to severe (substantia nigra, coeruleus/subcoeruleus complex, pedunculopontine nucleus, dorsal motor nucleus of vagus). Created in BioRender. Alpin, N. (2026) https://BioRender.com/7afori3.

**Table 2:** Distribution of α-synuclein pathology and degeneration severity in brainstem nuclei across synucleinopathies with RBD

Study	Sample	Clinical diagnosis	Nucleus	α-Synuclein	Neuronal loss severity
Uchiyama *et al.*^10^	*N* = 1	RBD	Substantia nigra	−	+++
			Locus coeruleus	−	+++
			Dorsal raphe nucleus	−	−
			Central raphe nucleus	−	−
			Pedunculopontine nucleus	−	−
			Nucleus gigantocellularis	−	−
			Ventral reticular nucleus	−	−
Schenck *et al.*^108^	*N* = 1	RBD + Ad	Substantia nigra	−	+
			Locus coeruleus	−	+++
			Pedunculopontine nucleus	−	↑
Benarroch *et al.*^110^	*N* = 4	RBD + MSA	Locus coeruleus	+	+++
			Dorsal raphe nucleus	+	−
			Medial raphe nucleus	+	−
			Pedunculopontine nucleus	+	+++
Boeve *et al.*^11^	*N* = 1	RBD	Substantia nigra	+	+
			Locus coeruleus	+	+
			Central raphe nucleus	+	+
			Pedunculopontine nucleus	+	+
			Nucleus gigantocellularis	+	+
			Dorsal motor nucleus of vagus	+	+
Iranzo *et al.*^106^	*N*_RBD_ _+_ _PD_ = 2 *N*_RBD_ _+_ _DLB_ = 1	RBD + PD/DLB	Substantia nigra	+	++ to +++
			Coeruleus/subcoeruleus complex	+	++
			Central raphe nucleus	+	+ to ++
			Pedunculopontine nucleus	+	+ to ++
			Nucleus gigantocellularis	+	+
			Dorsal motor nucleus of vagus	+	+++
Iranzo *et al.*^107^	*N* = 1	RBD + MCI	Substantia nigra	+	+ to ++
			Coeruleus/subcoeruleus complex	+	+
			Central raphe nucleus	+	+
			Pedunculopontine nucleus	+	++
			Nucleus gigantocellularis	+	+
			Dorsal motor nucleus of vagus	+	++
Mayà *et al.*^12^	*N* = 3	RBD	Substantia nigra	+	+ to ++
			Coeruleus/subcoeruleus complex	+	++
			Nucleus gigantocellularis	+	− to +

Summary of post-mortem findings on α-synuclein immunoreactivity and severity of neuronal loss in brainstem nuclei implicated in REM sleep regulation. The data highlight variability in the degree of degeneration across cases and diagnoses, underscoring the heterogeneity of brainstem pathology in α-synucleinopathies presenting with RBD. Note that no α-synuclein is reported in the Uchiyama *et al.*^10^ and Schenck *et al.*^108^ papers because α-synuclein antibodies were only developed later. α-Synuclein immunoreactivity is depicted as not present (−) or present (+). Severity of neuronal loss is represented by mild (+), moderate (++), and severe (+++). Note that ↑ refers to an increase in cell numbers. Ad, Alzheimer's disease; DLB, dementia with Lewy bodies; MCI, mild cognitive impairment; MSA, multiple system atrophy; PD, Parkinson's disease; RBD, REM sleep behaviour disorder.

### Neuroimaging studies

In contrast to post-mortem studies, neuroimaging studies can more easily attain larger sample sizes to investigate RBD in the absence of comorbid disease and offer the prospect of prognosis. Imaging studies of RBD to date have included structural, functional, diffusion, and dopamine transporter neuroimaging. Since neuroimaging studies on RBD vary in sample size, methodology, anatomical focus, and analysis techniques, findings from large multicentre cohorts generally provide the most robust evidence base for both early changes related to the primary pathophysiological mechanisms of RBD and later alterations related to phenoconversion and associated neurodegenerative processes. Additionally, structural lesion studies of secondary RBD may provide some of the clearest mechanistic insight into the minimal neuroanatomical circuitry sufficient to generate RSWA and DEB. Consequently, interpreting the findings in this section requires distinguishing abnormalities related to core REM sleep dysregulation from those associated with parallel or downstream neurodegeneration.

#### Structural neuroimaging

Structural neuroimaging studies have provided important anatomical information about the brain regions associated with RBD, particularly through analyses of focal lesion-associated RBD. Unlike isolated RBD case reports, which at post-mortem consistently already exhibit α-synuclein pathology, lesion-associated RBD may better isolate the core REM-regulating circuits necessary for RBD generation while minimizing the confounding effects of diffuse neurodegeneration.

Although specific nuclei affected by lesions and exact lesion boundaries are difficult to derive from MRI, case reports consistently attribute the clinical presentation of RBD to lesions in the pontine tegmentum (dorsal pons). Tippman-Peikert *et al*.^114^ described a case of acute-onset RBD in a 51-year-old woman with MS in remission, whose MRI showed the development of a large dorsal pontine plaque. Similarly, Plazzi and Montagna^46^ reported violent RBD episodes associated with a pontine lesion in probable MS, while Enriquez-Marulanda *et al.*^47^ described RBD as the presenting symptom of MS, with demyelinating lesions in the pontine tegmentum and bilateral cerebellar peduncles. However, RBD has also been reported in cases with larger lesions extending to the ventral and central pons,^48,115–118^ as well as the medulla and midbrain.^119^ The pontine area has also been implicated in RBD following ischaemic strokes and haemorrhages,^115–117,120–122^ cavernomas,^118^ tumours,^123^ and surgical interventions involving the pons.^124^

Lesion localization partially overlaps across REM dysregulation disorders. In an observational report of three cases with 37**–**50 years of history of narcolepsy with cataplexy, Plazzi *et al.*^125^ describe a finding of overlapping lesions in the central rostral pons in all cases. Cases with co-occurring narcolepsy and RBD, in turn, have been associated with lesions involving the dorsolateral midbrain and middle-to-caudal pontine tegmentum.^126,127^ Neighbouring pontomesencephalic structures may contribute differentially to REM-state stability, cataplexy, RSWA, and DEB. Comparative analyses on narcolepsy and RBD could be particularly interesting due to the opposing natures of cataplexy and RSWA, as well as their high rate of co-occurrence in individuals with narcolepsy.

Beyond focal lesions, volumetric MRI studies have identified cortical thinning and atrophy in isolated RBD, particularly involving the frontal,^128^ posterior parietal, temporal, and occipital cortices.^129^ This spatial pattern implies that neurodegeneration in α-synucleinopathy-associated RBD is not restricted to early brainstem sites but involves diffuse cortical regions even prior to clinical conversion. However, distinguishing whether these cortical abnormalities reflect mechanisms specific to RBD versus broader neurodegenerative processes or disease severity remains challenging. Supporting the concept of widespread vulnerability, a multicentre cohort of 182 isolated RBD patients identified that brain atrophy follows the expression pattern of synucleinopathy-associated genes, *SNCA* and *GBA*,^130^ linking cortical involvement in RBD to the molecular pathways of α-synuclein dysfunction and aggregation.

#### Functional neuroimaging

A recent synthesis of functional MRI (fMRI) studies in isolated RBD implicates consistent, albeit heterogeneous, alterations in functional connectivity across the basal ganglia, brainstem, and distributed cortical networks in isolated RBD, with longitudinal changes aligning with disease progression.^60^ Early resting-state fMRI studies in RBD patients focused on the association between RBD and PD, showing that altered nigrostriatal and nigrocortical connectivity may characterize the stages prior to motor impairment.^131–133^ Subsequent whole-brain analyses by Li *et al.*^134^ demonstrated reduced functional connectivity between the brainstem and posterior cerebellum, left temporal lobe, and anterior cingulate cortex. Additionally, in a whole-brain analysis with a complex network approach, Campabadal *et al.*^135^ showed that cortico-cortical connectivity is also reduced within parietal regions, which is consistent with prior studies that have reported changes in parietal cortical thickness in RBD.^129^ Whether these findings reflect RBD-specific dysfunction, broader neurodegenerative processes, or methodological differences between studies remains uncertain. Groenewald *et al.*^136^ also showed an increase in motor cortex perfusion during REM sleep in RBD episodes, with similar patterns of activation observed in the somatosensory and prefrontal cortices.

Complementary evidence comes from FDG-PET studies, which reveal distinct metabolic patterns in isolated RBD, characterized by regionally selective hypo- and hypermetabolism. Relative hypometabolism has been described in posterior cortical regions, including occipital, temporal, parietal, and cingulate cortices,^137–141^ with the involvement of precuneus and cuneus in isolated RBD cases with MCI.^139^ Interestingly, Orso *et al.*^142^ identified two different brain glucose metabolism patterns that could discriminate RBD phenoconversion to either PD or DLB. While both parkinsonism-first and dementia-first converters shared several metabolic changes, only dementia-first converters showed a metabolic decrease in the precuneus of the superior parietal lobule. Mattioli *et al.*^139^ further showed that relative hypometabolism is progressive through controls, isolated RBD cases, and finally isolated RBD cases with MCI, suggesting that FDG-PET may capture metabolic changes alongside disease progression and phenoconversion. These findings likely represent prognostic markers of phenoconversion and cognitive decline in RBD, rather than mechanisms directly associated with RSWA or DEB.

In contrast, hypermetabolism in isolated RBD has been reported in subcortical areas, including the brainstem, thalamus, cerebellum, putamen, sensorimotor areas, and hippocampus.^137–140,143^ This pattern has been interpreted as reflecting a combination of ongoing neurodegeneration and compensatory network activity. However, a longitudinal study by Kim *et al.*^143^ demonstrates that while the metabolic pattern can differentiate between isolated RBD and healthy controls at baseline, the pattern declines with longitudinal follow-up and cannot be used to predict phenoconversion reliably. These longitudinal changes were driven by attenuation of both posterior cortical hypometabolism and frontal and subcortical hypermetabolism, indicating that prodromal disease metabolic patterns are dynamic but not linearly progressive. Accordingly, FDG-PET findings may be useful as markers of evolving network dysfunction but do not always constitute robust predictors of phenoconversion trajectories. Thus, functional neuroimaging studies suggest that brain network dysfunction in RBD extends beyond subcortical regions and likely reflects parallel or downstream effects of an evolving neurodegenerative process.

#### Diffusion imaging

A large systematic synthesis of RBD neuroimaging studies highlights how diffusion-based imaging methods provide some of the most consistent in vivo evidence for early brainstem and white matter involvement in RBD.^144^ Early diffusion imaging studies in isolated RBD demonstrated decreased mean diffusivity in the pons, SN, internal capsule, and olfactory regions,^145^ consistent with compromised white matter integrity. Markers of loss of axonal integrity were also found in the left superior temporal lobule, pointing to early involvement of sensory processing and integration areas.^145^ Later investigations extended these findings to the mesencephalic tegmentum and rostral pons,^146^ and the SN,^147^ along with increased mean diffusivity indicative of tissue degradation in the pontine tegmentum.^146^ Since these regions overlap with pontomedullary REM sleep circuits, diffusion abnormalities in the pontine and mesencephalic tegmentum may relate more closely to primary RBD mechanisms, whereas the involvement of olfactory and sensory processing areas may reflect concomitant α-synuclein pathology.

Diffusion imaging studies have also suggested more extensive and diffuse neurodegenerative involvement in RBD.^148,149^ Olhauser *et al.*^150^ showed that patients with isolated RBD, relative to those with de novo PD, exhibit increased mean diffusivity in the major association and projection tracts, including the corpus callosum, internal and external capsules, superior and inferior longitudinal fasciculi, corticospinal tract, forceps major, and corona radiata. A recent multicentre study by Tremblay *et al.*^151^ showed disruptions in structural connectivity in frontal and posterior brain regions, and reductions in network efficiency in subcortical and sensorimotor areas. Compared with the more localized pontine and mesencephalic findings, these widespread alterations may be associated with the neurodegenerative processes underlying phenoconversion.

Interestingly, diffusion imaging studies have implicated specific regions in phenoconversion from RBD to α-synucleinopathies. The same multicentre study by Tremblay *et al.*^151^ suggested that local efficiency increases in the right supramarginal gyrus predicted phenoconversion from isolated RBD to DLB, but not PD. Another longitudinal multicentre study quantified free water (FW) from diffusion MRI and demonstrated higher FW in the NBM and posterior SN, but only in phenoconverters.^152^ Importantly, FW in the NBM specifically identified phenoconversion to DLB, but was not associated with the presence of isolated RBD. Therefore, diffusion imaging is likely to be better suited as a prognostic than diagnostic marker.

Diffusion imaging has also been used to probe glymphatic system function in isolated RBD. By assessing perivascular diffusion properties linked to CSF exchange, diffusion imaging provides indirect measures of glymphatic clearance. Emerging evidence suggests altered glymphatic function in isolated RBD, with reduced perivascular fluid transport and impaired CSF exchange,^153,154^ linking sleep-related physiological disruption to the altered clearance of aggregation-prone proteins such as α-synuclein. Although methodological differences among diffusion imaging studies warrant caution when comparing datasets, the findings support a model in which RBD involves abnormalities in both brainstem REM-sleep-regulating structures and their distributed connections.

#### Dopamine transporter imaging

Dopamine transporter (DAT) single-photon emission computer tomography (DAT-SPECT) studies indicate that RBD is characterized by subclinical dopaminergic denervation that does not yet reach the threshold for the emergence of motor impairment.^14,15^ Large international multicentre data show that subclinical dopaminergic dysfunction predicts phenoconversion risk in isolated RBD. In a coordinated analysis of 123I-FP-CIT SPECT scans from nine centres, patterns of reduced striatal DAT binding improved prediction of phenoconversion.^155^ Importantly, individuals with isolated RBD who had higher Mini-Mental State Examination (MMSE) scores and relatively preserved, symmetric caudate DAT availability were more likely to develop PD, while RBD patients with lower MMSE scores and greater asymmetry in caudate DAT availability were more likely to convert to DLB.^155^ Therefore, DAT-SPECT may help stratify future phenoconversion trajectories, particularly when combined with measures of cognitive function. Consistent with these findings, a meta-analysis demonstrates that tracer uptake at the level of the caudate is similar between isolated RBD and PD without RBD, while putaminal tracer uptake decreases progressively from healthy controls to isolated RBD and finally to PD patients with RBD.^144^ These results support the hypothesis that isolated RBD patients are on the path towards developing an α-synucleinopathy, and is further supported by two key observations: (i) RBD patients may exhibit a 20–30% loss of SN neurons without showing clinical parkinsonism,^107^ and (ii) motor symptoms typically appear when ∼50% of SN neurons are lost.^156^

Collectively, neuroimaging findings in RBD likely reflect a combination of REM-sleep-specific pathophysiological mechanisms and broader phenoconversion and neurodegenerative processes. Many imaging findings converge anatomically with the nuclei implicated by post-mortem studies and neurophysiological models of REM motor control, including the brainstem, basal ganglia, and cortical motor and associative regions. Alterations involving pontomedullary structures may relate more directly to RSWA and DEB, whereas widespread cortical, striatal, and metabolic abnormalities may instead reflect disease subtype, severity, and α-synucleinopathy progression. However, accurately distinguishing alterations specific to RBD pathophysiological mechanisms from those associated with parallel or downstream neurodegeneration remains challenging. Table 3 summarizes the proposed distinction between the two features in RBD.

**Table 3:** Proposed distinction between RBD-associated versus phenoconversion-associated neuroimaging features in RBD

Modality	RBD-associated	Phenoconversion-associated
Structural MRI	Pontine lesions^46–48,114–122^	Cortical atrophy^128–130^
Resting-state fMRI	Altered brainstem and REM-network functional connectivity^60,131–136^	Progressive network disintegration^60,131–135^
FDG-PET	Subcortical hypermetabolism^137–140,143^	Posterior cortical hypometabolism and longitudinal metabolic decline associated with MCI^137–142^
Diffusion imaging	Brainstem white matter disruption; reduced perivascular fluid transport and CSF exchange^145–147^	Supramarginal gyrus local efficiency increase predicting DLB; NBM and posterior SN FW increase predicting DLB^151–154^
DAT-SPECT	Early dopaminergic dysfunction^14,15^	Striatal asymmetry combined with MMSE scores predicting DLB versus PD^14,15,144,155^

Abbreviations: CSF, cerebrospinal fluid; DAT-SPECT, dopamine transporter single-photon emission computed tomography; DLB, dementia with Lewy bodies; FDG-PET, fluorodeoxyglucose positron emission tomography; fMRI, functional magnetic resonance imaging; FW, free water; MCI, mild cognitive impairment; MMSE, Mini-Mental State Examination; NBM, nucleus basalis of Meynert; PD, Parkinson's disease; RBD, rapid eye movement sleep behaviour disorder; REM, rapid eye movement; SN, substantia nigra.

### Pharmacological studies

Pharmacological studies complement post-mortem and neuroimaging approaches by providing indirect insight into the neurotransmitter systems implicated in REM sleep regulation in RBD. Currently, there are no pharmacological treatment options with high-level clinical trial evidence for RBD. Certain treatments have been shown to alleviate symptoms on a case-to-case basis but placebo-controlled trials have been negative. The pharmacological agents most associated with the triggering or amelioration of RBD symptoms include antidepressants, levodopa, melatonin, and benzodiazepines (Table 4; see Supplementary Table 1 for the complete version). Collectively, these pharmacological observations are consistent with the involvement of distributed monoaminergic, cholinergic, circadian, and GABAergic systems in RBD but they should be interpreted within the broader context of α-synucleinopathy-associated network dysfunction.

**Table 4:** Pharmacological agents associated with both triggering and amelioration of RBD symptoms

Pharmacological agent	Compound/method	References	Initial diagnosis	Effect on RBD symptoms	Proposed mechanism^a^
Antidepressants	SSRI, SNRI, bupropion pooled	McCarter *et al*.^157^	PTSD, MDD	Emergence of RSWA	Unmasking prodromal neurodegeneration or causing neurodegeneration
	SSRI, SNRI pooled	Lee *et al*.^158^	GAD, MDD, OCD, fibromyalgia, bipolar affective disorder, or other	Emergence of RSWA	Interference with monoamine activity during REM sleep
	Fluoxetine, paroxetine, citalopram, sertraline, venlafaxine, bupropion pooled	Winkelman *et al*.^49^	MDD, GAD	Emergence of RSWA	Influence on motor tone during REM sleep at brainstem or spinal levels
	Duloxetine	Tan *et al*.^159^	GAD	Increase in REM sleep latency and decrease of REM sleep time; RSWA, dream enactment behaviour	Serotonergic and norepinephrinergic influence on motor tone
Levodopa		Garcia-Borreguero *et al*.^160^	PD	Increased RBD frequency; increased phasic EMG activity	Progression of the disease rather than levodopa
		Tan *et al*.^161^	RBD-PD	Decreased RBD features	Suppression of REM sleep
Melatonin		Takeuchi *et al*.^162^	RBD (comorbidities unspecified)	Partial restoration of muscle atonia (decrease in tonic REM muscle activity)	Restoration of circadian desynchronization
	Chronobiotic protocol	Kunz *et al*.^163^	RBD	Substantial improvement of RBD symptoms	Resynchronisation of circadian rhythm
	Prolonged release	Jun *et al.*^164^	RBD	No improvement in frequency or severity of RBD symptoms	Unspecified
Cholinesterase inhibitors	Donepezil	Ringman & Simmons^165^	RBD-(Ad)	Marked improvement of RBD symptoms	Unspecified
	Rivastigmine	Brunetti *et al.*^166^	RBD-MCI	Marked reduction of RBD episodes	Modulation of muscle atonia during REM sleep, or restoration of cholinergic transmission in patients with existing cholinergic dysfunction
Benzodiazepines	Clonazepam	Schenck *et al*.^39,167^	RBD	Complete (77.2% of subjects) or partial (12.3% of subjects) control of dream enactment and altered dreams	Suppression of excessive phasic motor activity during REM sleep; modification of dream content
	Clonazepam	Li *et al*.^168^	RBD	Complete elimination of injurious or potentially injurious dream enactment behaviours in 66.7% of subjects	Modulation and reduction of fear response, inhibition of brainstem locomotor pattern generators, or activation of subcortical and cortico-limbic and paralimbic structures
	Clonazepam	McCarter *et al*.^169^	RBD, RBD-PD, RBD-MCI, RBD-MSA, RBD-probable DLB	Moderate or great level of improvement in 78%, complete suppression of RBD in 17%.	Promotion of GABAergic inhibition and reduction of REM %

^a^The mechanism suggested by the authors of the corresponding publication.

Ad, Alzheimer’s disease; DEB, dream enactment behaviour; DLB, dementia with Lewy bodies; GAD, Generalized anxiety disorder; LBD, Lewy body disease; MCI, Mild cognitive impairment; MDD, Major depressive disorder; MSA, multiple system atrophy; OCD, Obsessive-compulsive disorder; PD, Parkinson’s disease; PTSD, Post-traumatic stress disorder; RBD, REM sleep behaviour disorder; RSWA, REM sleep without atonia.

Note. Complete version of the table can be found in Supplementary Materials.

Antidepressants have been associated with RBD onset, with symptoms reported in up to 6% of users.^49–51^ A longitudinal study by Postuma *et al.*^170^ shows that antidepressant-associated RBD in older individuals often co-occurs with other prodromal features of neurodegeneration, including higher Unified Parkinson’s Disease Rating Scale (UPDRS) scores, abnormalities in olfaction and colour vision, and MCI. In these cases, antidepressants may have unmasked the prodromal stage of an underlying neurodegenerative process rather than have directly caused RBD. Younger-onset antidepressant-associated RBD may represent a distinct clinical phenotype. For instance, Zhang *et al.*^171^ demonstrated that sertraline administration to a younger, predominantly female population over 8 weeks led to increased RSWA, which resolved after drug discontinuation. Their findings suggest that antidepressants may also transiently disrupt REM motor control through monoaminergic mechanisms in the absence of neurodegeneration.

Dopaminergic dysfunction has likewise been implicated in RBD, although the effects of levodopa remain inconsistent across studies.^160,161,172,173^ Some studies report increased RBD frequency and phasic REM muscle activity in response to levodopa treatment, while others show improvements in symptoms.^162,163,173,174^ A large longitudinal study on 356 individuals with PD found that levodopa had no influence on REM sleep time,^174^ suggesting that any improvement in RBD symptoms is unlikely to be related to the suppression of REM sleep. These conflicting findings could reflect more complex effects on brainstem and basal ganglia circuitry involved in REM sleep motor control. For example, levodopa could differentially influence nocturnal motor regulation, dream-related activity or motor output gain depending on disease stage and dosing. However, these proposed circuit-level explanations remain speculative and have not been directly demonstrated in RBD.

Cholinergic and hypothalamic systems also modulate REM motor control in RBD. Melatonin, like levodopa, has shown varying efficacies in the treatment of RBD, with some studies reporting partial restoration of muscle atonia^162,163^ and others showing no significant benefit in randomized controlled trials.^164,175^ McGrane *et al*.^176^ critically reviewed that while some studies report a significant decrease in RSWA after melatonin treatment, RSWA may be an incidental finding and insufficient as a sole measure for comparison to clinical RBD. Cholinesterase inhibitors, including donepezil and rivastigmine, have also shown symptomatic improvement in RBD, particularly in patients with coexisting cognitive impairment.^165,166,177^ These effects may reflect enhanced cholinergic neurotransmission in patients with underlying cholinergic dysfunction, although such a proposed mechanism requires further validation.

Clonazepam has shown efficacy in reducing injurious DEB and nightmares in observational RBD cohorts.^39,168,169,178–182^ However, a 2019 double-blind clinical trial by Shin *et al*.^183^ reported no differences in RBD outcome after 4 weeks of treatment. One proposed explanation for its symptomatic effects is modulation of dream content, which tends to be unpleasant and violent in RBD. However, the relationship between changes in dream content and reductions in DEB remains uncertain, and evidence supporting this hypothetical mechanism is limited.

A 2025 consensus report from the international RBD study group recommended that antidepressants, including tricyclic, selective serotonin reuptake inhibitors (SSRIs), selective noradrenaline reuptake inhibitors (SNRIs), are discontinued, substituted, or at least reduced in dosage.^184^ The efficacy of melatonin, clonazepam, and some cholinergic and dopaminergic agents^167,185,186^ varies and their use may be limited by the risk of adverse effects such as gait imbalance.^184^ Developing a comprehensive theory on RBD pathogenesis would require understanding the exact pharmacological mechanisms through which these medications affect RBD symptoms.

### Genetic studies

Genetic studies of RBD complement pharmacological, neuroimaging, and post-mortem neuropathology approaches by identifying genetic risk factors shared with neurodegenerative diseases. They provide additional support for the hypothesis that RBD represents a biologically distinct and selectively vulnerable subtype within the α-synucleinopathy spectrum. The genetic architecture of RBD appears to preferentially implicate lysosomal and synaptic pathways through genes involved in proteostasis and vesicular trafficking. Several genes associated with PD or DLB concern synaptic and lysosomal function, including *GBA*,^185,186^  *SNCA*,^187–189^  *MAPT*,^190,191^  *LRRK2*^192^ and *TMEM175.*^193^ Genetic studies of RBD,^194–199^ including a genome wide association study,^37^ have implicated mutations in *GBA*,^197–200^  *SNCA*,^201^ SCARB2,^37^ and *TMEM175*,^37,194–199,202^ whereas other major PD-associated loci, such as *MAPT*^194^ and *LRRK2*^195^ show weaker or absent associations with RBD. These findings suggest that the pathological mechanisms underlying RBD may only partially overlap with those driving PD or DLB more broadly.

Importantly, differential associations at the *SNCA* locus may indicate region-specific vulnerability patterns that distinguish RBD from PD. The RBD-associated *SNCA* variant rs3756059 has been linked to reduced cortical expression of SNCA-AS1,^37^ an antisense RNA associated with α-synuclein regulation. Reduced expression of SNCA-AS1 may increase cortical susceptibility to α-synuclein accumulation and neurodegeneration, and could help explain the accelerated cognitive decline observed in individuals with co-occurring PD and RBD compared with those with PD alone.^203^ Additionally, mutations in *GBA* appear to be associated with faster progression from isolated RBD to PD,^200^ supporting the concept that genetic factors may influence not only susceptibility to RBD but also the phenoconversion trajectory and disease severity. RBD may be a biologically distinct α-synucleinopathy subtype with selective cortical vulnerability and autophagy-lysosomal pathway dysfunction.^37^ Pharmacologically, these findings generate interest in therapies targeting autophagy-lysosomal dysfunction and α-synuclein aggregation, particularly in *GBA*-associated disease. However, the discussed genetic variants are neither necessary nor sufficient for the development of RBD, and most individuals with PSG-confirmed RBD appear sporadic and lack clear family history.^204^

## Phenoconversion from RBD to α-synucleinopathies

Generally, there is a high risk of phenoconversion from RBD to α-synucleinopathies. Determining the absolute risk of phenoconversion, however, is complicated by α-synucleinopathy heterogeneity and variation across studies regarding definitions of outcome, follow-up time, and RBD onset.

Neurodegenerative diseases exhibit considerable heterogeneity regarding their onset and progression. Consequently, phenoconversion is more likely to be identified in RBD patients with more frequent and detailed assessments when early and subtle symptoms are detected. Patterns of phenoconversion vary and trajectories are likely to be determined by the direction of dysfunction beyond initially affected REM-regulating brainstem circuits. Differences in cortical involvement, SN cell loss, autonomic network involvement, and co-pathologies may influence whether individuals first present with parkinsonism, dementia, or dysautonomic phenotypes. However, studies assessing the phenoconversion from RBD to α-synucleinopathies often pool different α-synucleinopathies (i.e. PD, DLB and PDD) with overlapping symptoms together based on the onset of their shared symptom of parkinsonism. Although such pooling efficiently communicates the overall risk of RBD patients developing a neurodegenerative disease, it overlooks the distinct clinical trajectories of each disease.

Other factors that complicate the risk assessment of phenoconversion are variable prodromal period lengths among α-synucleinopathies and variable follow-up examinations among studies. Rather than reflecting a uniform prodromal phase, phenoconversion from RBD to α-synucleinopathies follows heterogenous trajectories, with risk increasing over time and approaching 90% in long-term follow-up.^33–35,205^ In a 10-year prospective cohort with annual follow-up evaluations, Postuma *et al.*^9^ showed that 30% of RBD patients convert to PD or dementia at 3 years from the initial RBD diagnosis, rising to 66% at 7.5 years. Many studies have corroborated this and show phenoconversion rates ranging from 20.5% to 27.3% at the 2–4-year mark.^140,168,206^ A large longitudinal multicentre synthesis that includes many previously published cohorts shows that at 13 years, the proportion of converters ranges from 73.5% to 82%.^205^ The phenoconversion risk therefore increases over time and, with sufficiently long follow-up, most patients develop an α-synucleinopathy, although individual trajectories can vary significantly.

Phenoconversion studies rarely distinguish between the onset of RBD and the time of clinical diagnosis. Diagnostic delays of up to 9 years have been described in RBD, suggesting that the true length of the prodromal period may be even a decade longer than the literature estimates.^207,208^ For example, in a cohort of 29 older men initially diagnosed with isolated RBD, Schenck *et al.*^39^ demonstrated that 38% of patients developed PD at only 3.7 years after the diagnosis, but their RBD symptoms had begun an average of 9 years earlier. In an update 16 years later, the authors noted that 81% of the 26 patients who were not lost to follow-up had eventually developed an α-synucleinopathy.^33^ Thus, calculating time to phenoconversion from symptom onset rather than clinical diagnosis generally results in lower estimates of cumulative phenoconversion during the first decade. Figure 4 illustrates how estimates of neurodegenerative-disease-free survival differ according to the chosen time reference. Similar lower rates of phenoconversion in the first decade have also been reported for three other cohorts: Zhou *et al.*^210^ (28% after 9 years from onset), Arnulf *et al.*^211^ (37.5% after 9 years from onset and 3 years from diagnosis), and Youn *et al.*^209^ (24% after 10 years from onset), although estimates among the cohorts vary depending on factors such as the recruitment base and stringency of diagnostic criteria.^196,212^

**Figure 4:**
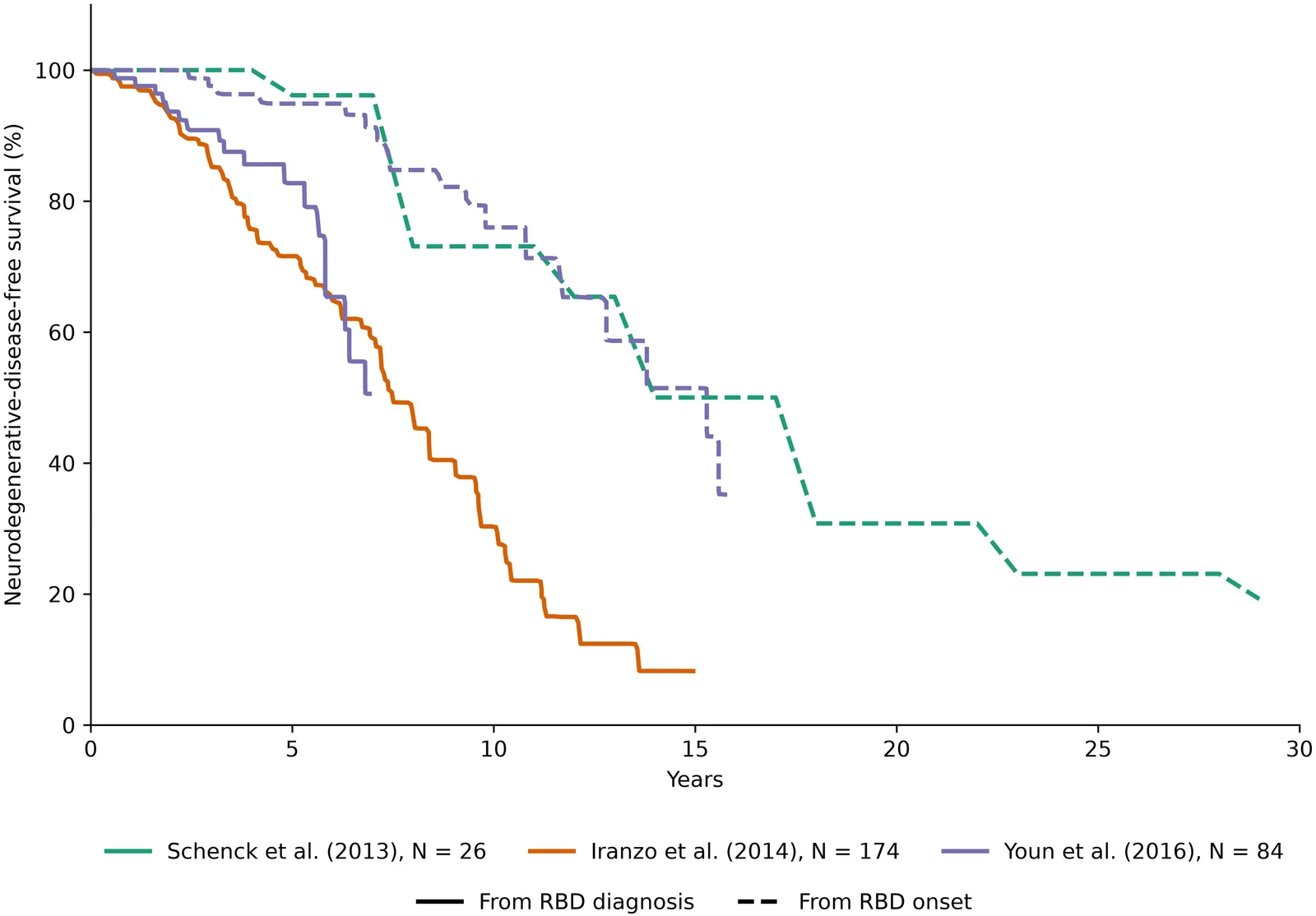
**Effect of the reference time point on Kaplan–Meier estimates of neurodegenerative-disease-free survival in RBD.** Four Kaplan–Meier curves are presented as illustrative examples from three independent published RBD cohort studies. Curves from Iranzo *et al*.^34^ (orange, *N* = 174) and Youn *et al*.^209^ (violet, *N* = 84) were digitized from published Kaplan–Meier figures using WebPlotDigitizer v5.2, whereas the curve for the longer-term cohort study by Schenck *et al*.^33^ (green, *N* = 26) was approximated from published summary statistics (median 14 years, mean 14.2 years, standard deviation 6.2 years). Neurodegenerative-disease-free survival represents the proportion of individuals with RBD who had not yet developed an α-synucleinopathy. Survival was estimated from either the time of RBD diagnosis (solid lines) or the reported onset of RBD symptoms (dashed lines). Youn *et al*.^209^ curves reproduced from *Progression of cognitive impairments in idiopathic REM sleep behaviour disorder*, Soyoung Youn, Tae Kim, In-Young Yoon, Jahyun Jeong, Hye Young Kim, Ji Won Han, Jong-Min Kim, and Ki Woong Kim, *Journal of Neurology, Neurosurgery & Psychiatry,* Volume 87, 890–896, 2016, with permission from BMJ Publishing Group Ltd.

Collectively, individual studies of phenoconversion vary widely in sample size, whether they make a distinction between RBD onset and time of clinical diagnosis, and outcome definitions. Nonetheless, a 2019 meta-analysis that synthesized longitudinal cohorts published up to 2017^35^ shows that the risk of developing a neurodegenerative disease was 33.5% at 5 years, 82.4% at 10.5 years, and a very high 96.6% at 14 years, with the majority of RBD patients converting to PD (43%), followed by DLB (25%).

### Peripheral α-synuclein as a phenoconversion biomarker

The high rate of phenoconversion from RBD to α-synucleinopathies suggests that RBD is a strong predictor of neurodegenerative disease, and that biomarkers predictive of α-synucleinopathies may also be predictive of RBD. The most prominent biomarker candidate is α-synuclein itself, which exhibits prion-like self-propagating and aggregating behaviour in the misfolded state.^213–215^ Since RBD is thought to reflect the body-first subtype of the body-first versus brain-first hypothesis, individuals with RBD (i.e. body-first cases) may be more likely to have misfolded α-synuclein in peripheral tissues such as the skin, whereas those with a brain-first pattern at comparable disease stages may have limited or absent peripheral pathology.

Detecting biomarkers in relatively accessible peripheral tissues has been a long-standing goal towards early disease identification. A biophysical protein amplification technique known as the seed amplification assay (SAA) is often used to detect trace amounts of misfolded α-synuclein in biological samples and has received considerable attention regarding the early identification of isolated RBD patients at risk for phenoconversion. SAA has been applied to olfactory mucosa,^216,217^ skin,^32,217^ and CSF samples^30–32,218,219^ of patients with isolated RBD or established α-synucleinopathies.

In a recent meta-analysis, Iftikhar *et al*.^220^ reported that SAA can rather reliably detect α-synuclein aggregation in RBD biosamples, with sensitivities and specificities reported highest in CSF and dermal biopsies (combined sensitivity and specificity of 78% and 99%, respectively). Across studies, ∼73% of individuals with isolated RBD are SAA-positive at baseline,^30–32,216–219^ and longitudinal follow-up suggests that SAA may detect pathological α-synuclein up to 9 years before clinical phenoconversion.^31,218^

Both technical and biological factors influence why not all converters are always baseline-SAA-positive. SAA protocols vary among laboratories regarding reagent preparation, reaction conditions, and readout parameters. Moreover, SAA primarily yields a binary readout on whether α-synuclein in a biosample can seed amplification. Ongoing efforts are focused on identifying whether specific SAA kinetic parameters (e.g. lag time, peak rate of fluorescence, maximum fluorescence intensity) can predict RBD phenoconversion to specific α-synucleinopathies.

While SAA may be able to indicate whether an α-synucleinopathy is likely, SAA status should be interpreted in light of other measures that may offer complementary and sometimes superior information. For example, in the Liguori *et al.*^32^ cohort, direct detection of phosphorylated α-synuclein from dermal biopsies via immunofluorescence staining provided better diagnostic accuracy compared with dermal SAA. Moreover, quantitative motor assessments can detect subtle changes in balance, motor planning and execution, which may be more predictive of phenoconversion to dementia than to parkinsonism.^205^ Such distinctions are informative since RBD patients who develop PD have proportionally more periods of tonic RSWA than those who develop DLB,^221^ whereas DEB seems to be more frequent in PDD/DLB than in PD.^222^

The phenoconversion from RBD to dementia versus parkinsonism could be related to the spread of α-synuclein pathology upwards to the cortex before the SN (e.g. from olfactory regions to the mediodorsal thalamic nucleus and then the cortex^223^). Dementia-first (versus parkinsonism-first) RBD phenoconverters tend to have a longer prodromal stage, which may reflect a different degree of plasticity in regions outside the midbrain.^224^ Concomitant pathology related to Alzheimer’s disease (cortical β-amyloid and tau pathology) in RBD cases may also trigger cortical dysfunction or neurodegeneration and result in a dementia-first phenotype independent of or together with α-synuclein pathology.^225^ A recent study demonstrated that combining 4R-tau dermal SAA with α-synuclein dermal SAA improves differentiation of individuals with progressive supranuclear palsy (PSP) from those with PD or MSA.^226^ This combined approach may help address the common problem of misdiagnosis during the early stages of disease, and similar investigations in RBD cohorts could improve the prediction of subsequent disease trajectories. Therefore, SAA may complement neuroimaging, neuropathological, and motor assessments in the identification of individuals with RBD who are at risk for developing dementia, parkinsonism, or autonomic dysfunction first.

### RBD remission

Unless secondary to conditions such as brainstem lesions, narcolepsy or antidepressant use, RBD is typically progressive rather than reversible. However, there are reports of RBD symptom fluctuation and even remission in established α-synucleinopathies. Ye *et al.*^203^ showed that at the fourth year of follow-up, 17.6% of individuals with de novo PD and RBD at baseline were free of RBD symptoms at the follow-up. These individuals with RBD remission demonstrated faster deterioration of motor function, whereas individuals with persistent RBD since the initial consultation had a faster rate of cognitive decline.^203^ This 17.6% remission rate is more modest than the earlier reported rate of 33% by Gjerstad *et al.*^227^ at 4 years from diagnosis.^227^ However, both studies determined RBD diagnosis from a self-report questionnaire rather than PSG monitoring or consultation with bed partners and may not accurately represent the true frequency of RBD. Notably, in established PD cases, RBD remission rates remain substantially lower than progression rates.

The mechanisms that underlie RBD remission remain unresolved. Hypotheses such as the body-first versus brain-first model do not account for RBD remission, and instead assume that RBD reflects irreversible brainstem involvement once present. Cases of remission could be due to endogenous compensatory functions or the effects of medications that help to control symptoms (Table 4). An alternative hypothesis is that progression of neurodegeneration alters REM sleep architecture and dream-related neural activity,^228^ possibly reducing the complexity, vividness, and behavioural expression of dream-related mentation and thereby resulting in fewer noticeable RBD symptoms. However, this interpretation warrants caution, as persistence or remission of RBD symptoms may not necessarily mirror other neurodegenerative features such as cognitive decline. Ye *et al.*^203^ demonstrated that individuals with stable RBD exhibited faster cognitive decline and parahippocampal grey matter atrophy, suggesting that REM dysregulation and cognitive deterioration may reflect at least partially dissociable processes. Misdiagnosis should also be considered a possible explanation for apparent RBD remission, given that many RBD studies, especially retrospective ones, have included non-PSG-confirmed cases.

## Future directions

We have identified several key areas where further investigation could refine our understanding of the role of RBD in the pathogenesis of α-synucleinopathies. Based on the evidence presented, we propose several promising directions for future research that may help clarify the pathophysiology of RBD and the mechanisms underlying phenoconversion:

1. **REM sleep microstructure**
  The microstructure of REM sleep is complex and often overlooked. Valomon *et al.*^69^ observed a reduction in differentiation between tonic and phasic REM sleep in RBD patients, and multiple studies have reported that DEB can occur across sub-stages, despite evidence that there should be prominent muscle atonia during tonic REM sleep.^64–68^ Further research on the brainstem structures regulating transitions between these two REM microstates, particularly in RBD cohorts, is encouraged.
2. **Neuropathological insights**
  Since RBD is not a terminal condition, brain donations from individuals with isolated RBD are very rare, limiting their availability for brain bank studies. To address this, examining the neuropathology of PD patients with and without RBD, alongside a well-matched control group, could help to distinguish the structures implicated in RBD versus PD alone. Moreover, post-mortem investigations of individual nuclei within the REM sleep regulatory network could help clarify why, for instance, DBS of the PPN induces muscle atonia in PD but not in RBD.^94,95^
3. **Longitudinal Diffusion Tensor Imaging (DTI)**
  DTI can provide valuable information about the neurodegenerative patterns in RBD, but its application has been limited by variability in acquisition and analysis methods. Standardizing DTI protocols and analyses across studies would augment the reliability of findings in relation to changes in RBD and help clarify the true sequence of neuroanatomical changes throughout the process of phenoconversion.
4. **Longitudinal biomarker studies**
  Longitudinal cohorts enable tracking of changes in molecular, imaging, and physiological markers in RBD patients. Correlating clinical symptoms with SAA findings, amyloid/tau/neurodegeneration biomarkers, and peripheral immune activation over time is crucial for mapping disease progression and clarifying the dynamics of phenoconversion and the different trajectories from RBD to α-synucleinopathies.
5. **Comparative pathology: narcolepsy and RBD**
  A systematic comparison of narcolepsy and RBD pathology could reveal shared or distinct mechanisms related to their respective disruptions in REM sleep regulation. Narcolepsy type 1 (NT1) is characterized by orexin deficiency and profound REM dysregulation,^229^ while narcolepsy type 2 (NT2) lacks orexin loss but shares some of the clinical features with NT1.^230^ Comparing RBD with NT1 and NT2 may uncover convergent pathways or unique vulnerabilities that predispose individuals to α-synucleinopathies.

## Conclusions

RBD is recognized as the earliest and most specific prodromal manifestation of α-synucleinopathies. However, despite substantial advances in neuropathology, neuroimaging, and biomarker research, the mechanisms underlying RSWA and DEB are not completely understood. A major challenge in defining RBD-specific mechanisms is that many individuals with isolated RBD already exhibit evidence of an evolving α-synucleinopathy, making it difficult to distinguish changes related directly to REM sleep dysregulation from broader neurodegenerative changes associated with phenoconversion.

Across modalities, evidence consistently implicates distributed pontomedullary and mesencephalic structures in the generation of RSWA and DEB. These structures include the pontine tegmentum, ventromedial medulla, MLR, and their associated inhibitory and locomotor pathways. Lesion-associated RBD cases may provide some of the clearest mechanistic evidence for the minimal circuitry sufficient to generate REM sleep motor dysregulation, whereas widespread brainstem and limbic α-synuclein pathology and cortical, striatal, metabolic, and connectivity findings in isolated RBD cohorts likely reflect evolving α-synucleinopathy-related neurodegeneration, compensatory functions, and phenoconversion processes. Importantly, RSWA and DEB may only partially overlap mechanistically, which may contribute to the marked clinical heterogeneity of RBD. Accordingly, not all abnormalities observed in isolated RBD should be interpreted as primary drivers of RBD itself.

Taken together, findings across modalities suggest that RBD emerges from dynamic interactions between the dysfunction of REM sleep circuits that are selectively vulnerable in α-synucleinopathies and evolving network changes. Figure 5 presents a conceptual framework integrating REM sleep dysregulation, α-synucleinopathy-related network dysfunction, and phenoconversion trajectories in RBD. Future prospective longitudinal studies incorporating detailed clinical and neurophysiological characterization of individuals with isolated RBD, multimodal neuroimaging, quantitative neuropathology, molecular biomarkers, and comparative analyses across lesion-, narcolepsy-, antidepressant-, and α-synucleinopathy-associated RBD will be essential for disentangling mechanisms specific to RBD from those associated with parallel or downstream neurodegeneration. Such approaches may ultimately improve early phenoconversion stratification and help develop targeted disease-modifying interventions during prodromal and early stages of α-synucleinopathies.

**Figure 5:**
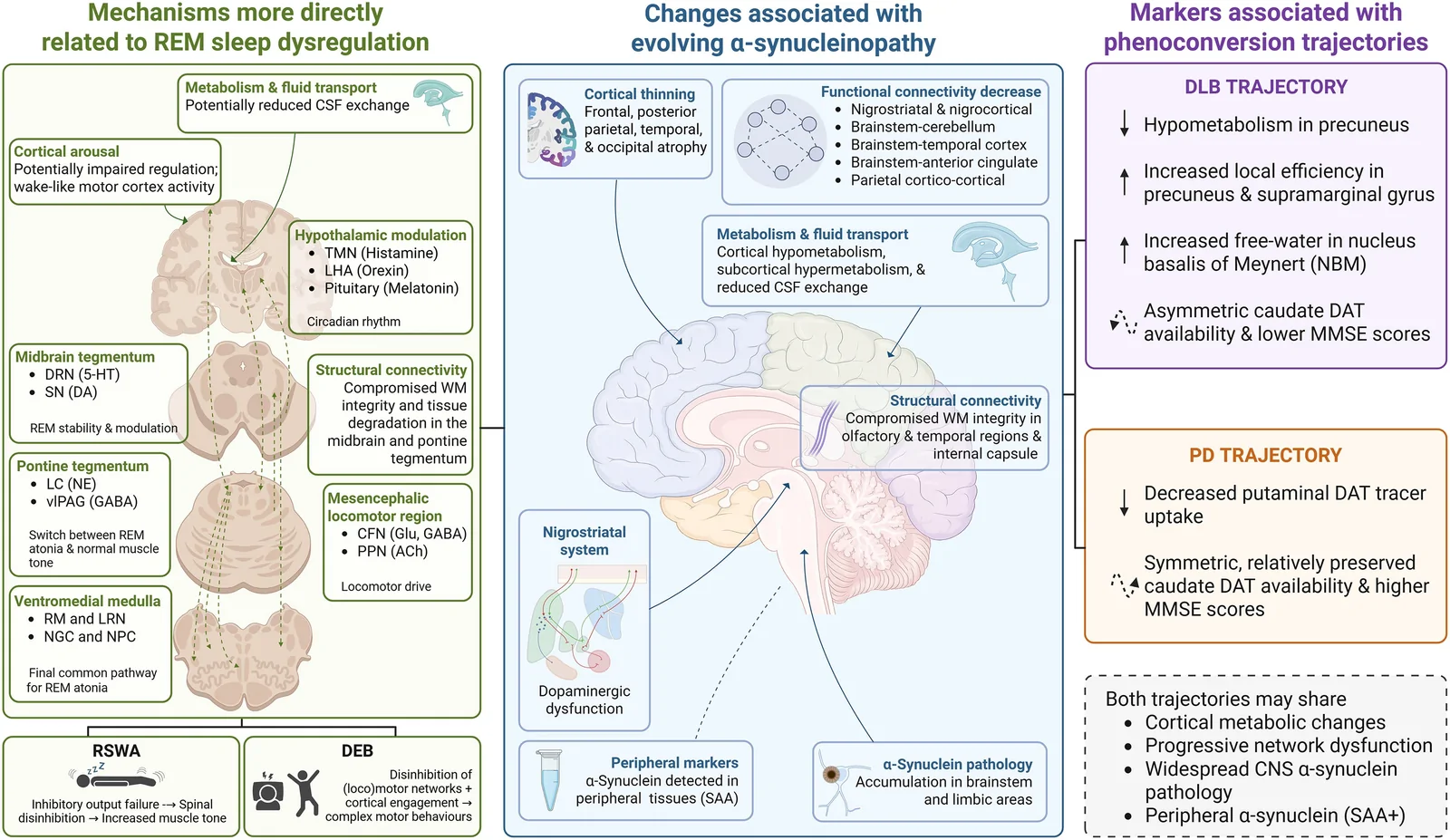
**Proposed framework linking REM sleep dysregulation, evolving α-synucleinopathy-related network dysfunction, and phenoconversion trajectories in RBD.** Left: Areas and mechanisms more directly related to REM dysregulation, including circuits contributing to RSWA and DEB. Centre: Widespread cortical, metabolic, connectivity, and biomarker abnormalities identified in isolated RBD cohorts, many of which may reflect broader neurodegenerative progression rather than primary RBD mechanisms. Right: Imaging and biomarker features associated with differential phenoconversion trajectories towards DLB or PD. The framework emphasizes that pontomedullary REM-regulating network dysfunction may contribute more directly to RSWA and DEB, whereas cortical and limbic abnormalities likely reflect downstream α-synucleinopathy processes and compensatory mechanisms. Created in BioRender. Alpin, N. (2026) https://BioRender.com/h38k5nc.

## Supplementary Material

fcag388_Supplementary_Data

## Data Availability

Data sharing is not applicable to this article as no new data were created or analysed in this study.
