# Supplementary material for: Pathophysiology of REM sleep behaviour disorder
Source: Brain Commun. 2026 Oct 15;8(6):fcag388. doi: 10.1093/braincomms/fcag388 (PMC13739046; doi:10.1093/braincomms/fcag388)
Supplement: Supplementary file 1 [file fcag388_supplementary_data.pdf]

Supplementary Table 1 Pharmacological agents associated with both triggering and amelioration of RBD symptoms

Pharmacological agent	Compound/method	Reference	Initial diagnosis	Effect on RBD symptoms	Proposed mechanisma
Antidepressants	SSRI, SNRI, bupropion pooled	McCarter et al.[1]	PTSD, MDD	Emergence of RSWA	Unmasking prodromal neurodegeneration or causing neurodegeneration
	SSRI, SNRI pooled	Lee et al.[2]	GAD, MDD, OCD, fibromyalgia, bipolar affective disorder, or other	Emergence of RSWA	Interference with monoamine activity during REM sleep
	Unspecified	Postuma et al.[3]	RBD	Increased phasic REM sleep EMG activity	Underlying neurodegenerative disease
	Sertraline	Zhang et al.[4]	MDD	Increased tonic and phasic RSWA	Unspecified
	Paroxetine	Parish et al.[5]	MDD	Emergence of clinical RBD	Unspecified
	Fluoxetine, paroxetine, citalopram, sertraline, venlafaxine, bupropion pooled	Winkelman et al.[6]	MDD, GAD	Emergence of RSWA	Influence on motor tone during REM sleep at brainstem or spinal levels
	Duloxetine	Tan et al.[7]	GAD	Increase in REM sleep latency and decrease of REM sleep time; RSWA, dream enactment behaviour	Serotonergic and norepinephrinergic influence on motor tone
Levodopa		Garcia-Borreguero et al.[8]	PD	Increased RBD frequency; increased phasic EMG activity	Progression of the disease rather than levodopa
		Tan et al.[9]	RBD-PD	Decreased RBD features	Suppression of REM sleep
		Yamauchi et al.[10]	Diffuse LBD	Improved RBD symptoms	Suppression of REM sleep
		Cao et al.[11]	PD ± RBD	Increased RBDSQ scores	D1 receptor activation; dopaminergic system may be more important in RBD development after PD onset than in the development of RBD before PD onset
Melatonin		Takeuchi et al.[12]	RBD (comorbidities unspecified)	Partial restoration of muscle atonia (decrease in tonic REM muscle activity)	Restoration of circadian desynchronization

	Chronobiotic protocol	Kunz et al.[13]	RBD	Substantial improvement of RBD symptoms	Resynchronisation of circadian rhythm
	Prolonged release	Jun et al.[14]	RBD	No improvement in frequency or severity of RBD symptoms	Unspecified
		Gilat et al.[15]	PD-RBD	No effect on RBD symptoms	Unspecified
Cholinesterase inhibitors	Donepezil	Ringman & Simmons[16]	RBD-(AD)	Marked improvement of RBD symptoms	Unspecified
	Rivastigmine	Di Giacopo et al.[17]	PD-RBD	Significant reduction of mean frequency of RBD episodes	Unspecified
	Rivastigmine	Brunetti et al.[18]	RBD-MCI	Marked reduction of RBD episodes	Modulation of muscle atonia during REM sleep, or restoration of cholinergic transmission in patients with existing cholinergic dysfunction
Benzodiazepines	Clonazepam	Schenck et al.[19,20]	RBD	Complete (77.2% of subjects) or partial (12.3% of subjects) control of dream enactment and altered dreams	Suppression of excessive phasic motor activity during REM sleep; modification of dream content
	Clonazepam	Li et al.[21]	RBD	Complete elimination of injurious or potentially injurious dream enactment behaviours in 66.7% of subjects	Modulation and reduction of fear response, inhibition of brainstem locomotor pattern generators, or activation of subcortical and corticolimbic and paralimbic structures
	Clonazepam	Fernandez-Arcos et al.[22]	RBD	Complete elimination of RBD in 92 (55.1%), partial in 52 (31.1%), no effect in 4 (2.4%)	Unspecified
	Clonazepam	Sunwoo et al.[23]	RBD	Improvement of RBD symptoms in 33 (34.4%) subjects	Unspecified
	Clonazepam	Wang et al.[24]	RBD	Complete elimination of RBD in 118 (88.7%) subjects; residual injurious behaviours in 11 (11.3%) subjects	Unspecified

Clonazepam	Lee et al.[25]	RBD	Positive treatment response in 137 (93.1%) subjects; no response in 10 (6.9%)	Unspecified
Clonazepam	Wing et al.[26]	RBD, RBD-PD, RBD-dementia	Significant reduction in sleep-related injuries (pretreatment 80.8% vs post-treatment 5.6%, $p < 0.05$)	Unspecified
Clonazepam	McCarter et al.[27]	RBD, RBD-PD, RBD-MCI, RBD-MSA, RBD-probable DLB	Moderate or great level of improvement in 78%, complete suppression of RBD in 17%.	Promotion of GABAergic inhibition and reduction of REM %
Clonazepam	Shin et al.[28]	PD-RBD	No effect	Unspecified

[a]The mechanism suggested by the authors of the corresponding publication.
AD, Alzheimer's disease; DEB, dream enactment behaviour; DLB, dementia with Lewy bodies; GAD, Generalized anxiety disorder; LBD, Lewy body disease; MCI, Mild cognitive impairment; MDD, Major depressive disorder; MSA, multiple system atrophy; OCD, Obsessive-compulsive disorder; PD, Parkinson's disease; PTSD, Post-traumatic stress disorder; RBD, REM sleep behaviour disorder; RSWA, REM sleep without atonia.

References

1. McCarter SJ, St. Louis EK, Sandness DJ, et al. Antidepressants Increase REM Sleep Muscle Tone in Patients with and without REM Sleep Behavior Disorder. *Sleep*. Published online June 1, 2015. doi:10.5665/sleep.4738

2. Lee K, Baron K, Soca R, Attarian H. The Prevalence and Characteristics of REM Sleep without Atonia (RSWA) in Patients Taking Antidepressants. *Journal of Clinical Sleep Medicine*. 2016;12(03):351-355. doi:10.5664/jcsm.5582

3. Postuma RB, Gagnon JF, Tuineaig M, et al. Antidepressants and REM Sleep Behavior Disorder: Isolated Side Effect or Neurodegenerative Signal? *Sleep*. 2013;36(11):1579-1585. doi:10.5665/sleep.3102

4. Zhang B, Hao Y, Jia F, et al. Sertraline and rapid eye movement sleep without atonia: An 8-week, open-label study of depressed patients. *Prog Neuropsychopharmacol Biol Psychiatry*. 2013;47:85-92. doi:10.1016/j.pnpbp.2013.08.010

5. Parish JM. Violent dreaming and antidepressant drugs: or how paroxetine made me dream that I was fighting Saddam Hussein. *J Clin Sleep Med*. 2007;3(5):529-531.

6. Winkelman JW, James L. Serotonergic Antidepressants are Associated with REM Sleep Without Atonia. *Sleep*. 2004;27(2):317-321. doi:10.1093/sleep/27.2.317

7. Tan L, Zhou J, Yang L, et al. Duloxetine-induced rapid eye movement sleep behavior disorder: a case report. *BMC Psychiatry*. 2017;17(1):372. doi:10.1186/s12888-017-1535-4

8. Garcia-Borreguero D, Caminero AB, de la Llave Y, et al. Decreased phasic EMG activity during rapid eye movement sleep in treatment-naïve Parkinson's disease: Effects of treatment with levodopa and progression of illness. *Movement Disorders*. 2002;17(5):934-941. doi:10.1002/mds.10233

9. Tan A, Salgado M, Fahn S. Rapid eye movement sleep behavior disorder preceding Parkinson's disease with therapeutic response to levodopa. *Movement Disorders*. 1996;11(2):214-216. doi:10.1002/mds.870110216

10. Yamauchi K, Takehisa M, Tsuno M, et al. Levodopa improved rapid eye movement sleep behavior disorder with diffuse Lewy body disease. *Gen Hosp Psychiatry*. 2003;25(2):140-142. doi:10.1016/S0163-8343(03)00003-3

11. Cao R, Ma R, Wang K, Hu P. Association Between Dopaminergic Medications and the Evolution of REM Sleep Behavior Disorder in Parkinson's Disease. *Front Neurol*. 2022;13. doi:10.3389/fneur.2022.880583

12. Takeuchi N, Uchimura N, Hashizume Y, et al. Melatonin therapy for REM sleep behavior disorder. *Psychiatry Clin Neurosci*. 2001;55(3):267-269. doi:10.1046/j.1440-1819.2001.00854.x

13. Kunz D, Stotz S, Bes F. Treatment of isolated REM sleep behavior disorder using melatonin as a chronobiotic. *J Pineal Res*. 2021;71(2). doi:10.1111/jpi.12759

14. Jun J, Kim R, Byun J, et al. Prolonged–release melatonin in patients with idiopathic <scp>REM</scp> sleep behavior disorder. *Ann Clin Transl Neurol*. 2019;6(4):716-722. doi:10.1002/acn3.753

15. Gilat M, Coeytaux Jackson A, Marshall NS, et al. Melatonin for Rapid Eye Movement Sleep Behavior Disorder in Parkinson's disease: A Randomised Controlled Trial. *Movement Disorders*. 2020;35(2):344-349. doi:10.1002/mds.27886

16. Ringman JM, Simmons JH. Treatment of REM sleep behavior disorder with donepezil: A report of three cases. *Neurology*. 2000;55(6):870-871. doi:10.1212/WNL.55.6.870

17. Di Giacopo R, Fasano A, Quaranta D, Marca G Della, Bove F, Bentivoglio AR. Rivastigmine as alternative treatment for refractory REM behavior disorder in Parkinson's disease. *Movement Disorders*. 2012;27(4):559-561. doi:10.1002/mds.24909

18. Brunetti V, Losurdo A, Testani E, et al. Rivastigmine for Refractory REM Behavior Disorder in Mild Cognitive Impairment. *Curr Alzheimer Res*. 2014;11(3):267-273. doi:10.2174/1567205011666140302195648

19. Schenck CH, Bundlie SR, Mahowald MW. Delayed emergence of a parkinsonian disorder in 38% of 29 older men initially diagnosed with idiopathic rapid eye movement sleep behavior disorder. *Neurology*. 1996;46(2):388-393. doi:10.1212/WNL.46.2.388

20. Schenck CH, Mahowald MW. Polysomnographic, neurologic, psychiatric, and clinical outcome report on 70 consecutive cases with REM sleep behavior disorder (RBD): sustained clonazepam efficacy in 89*5% of 57 treated patients. *Cleve Clin J Med*. 1990;57 Suppl.:S9-S23.

21. Li SX, Lam SP, Zhang J, et al. A prospective, naturalistic follow-up study of treatment outcomes with clonazepam in rapid eye movement sleep behavior disorder. *Sleep Med*. 2016;21:114-120. doi:10.1016/j.sleep.2015.12.020

22. Fernández-Arcos A, Iranzo A, Serradell M, Gaig C, Santamaria J. The Clinical Phenotype of Idiopathic Rapid Eye Movement Sleep Behavior Disorder at Presentation: A Study in 203 Consecutive Patients. *Sleep*. 2016;39(1):121-132. doi:10.5665/sleep.5332

23. Sunwoo JS, Kim YJ, Byun JI, et al. Comorbid Depression Is Associated with a Negative Treatment Response in Idiopathic REM Sleep Behavior Disorder. *Journal of Clinical Neurology*. 2020;16(2):261. doi:10.3988/jcn.2020.16.2.261

24. Wang J, Liu Y, Chau SWH, et al. Residual Injurious Symptoms and Its Association With Neurodegenerative Outcomes in Idiopathic Rapid Eye Movement Sleep Behavior Disorder: A Retrospective, Longitudinal <scp>Follow-up</scp> Study. *Movement Disorders*. 2020;35(11):2077-2085. doi:10.1002/mds.28210

25. Lee HJ, Choi H, Yoon IY. Age of Diagnosis and Comorbid PLMD Predict Poor Response of REM Behavior Disorder to Clonazepam. *J Geriatr Psychiatry Neurol*. 2021;34(2):142-149. doi:10.1177/0891988720915517

26. Wing YK, Lam SP, Li SX, et al. REM sleep behaviour disorder in Hong Kong Chinese: clinical outcome and gender comparison. *J Neurol Neurosurg Psychiatry*. 2008;79(12):1415-1416. doi:10.1136/jnnp.2008.155374

27. McCarter SJ, Boswell CL, St. Louis EK, et al. Treatment outcomes in REM sleep behavior disorder. *Sleep Med*. 2013;14(3):237-242. doi:10.1016/j.sleep.2012.09.018

28. Shin C, Park H, Lee WW, Kim HJ, Kim HJ, Jeon B. Clonazepam for probable REM sleep behavior disorder in Parkinson's disease: A randomized placebo-controlled trial. *J Neurol Sci*. 2019;401:81-86. doi:10.1016/j.jns.2019.04.029
